# Functional and Predictive Structural Characterization of WRINKLED2, A Unique Oil Biosynthesis Regulator in Avocado

**DOI:** 10.3389/fpls.2021.648494

**Published:** 2021-06-08

**Authors:** Jyoti R. Behera, Md. Mahbubur Rahman, Shina Bhatia, Jay Shockey, Aruna Kilaru

**Affiliations:** ^1^Department of Biological Sciences, East Tennessee State University, Johnson City, TN, United States; ^2^Department of Biological Systems Engineering, Virginia Tech, Blacksburg, VA, United States; ^3^United States Department of Agriculture, Agricultural Research Service, New Orleans, LA, United States

**Keywords:** AP2 domain, *Arabidopsis thaliana*, fatty acids, mesocarp, *Persea americana*, transcription factor, triacylglycerol, WRINKLED1

## Abstract

WRINKLED1 (WRI1), a member of the APETALA2 (AP2) class of transcription factors regulates fatty acid biosynthesis and triacylglycerol (TAG) accumulation in plants. Among the four known *Arabidopsis WRI1* paralogs, only *WRI2* was unable to complement and restore fatty acid content in *wri1-1* mutant seeds. Avocado (*Persea americana*) mesocarp, which accumulates 60-70% dry weight oil content, showed high expression levels for orthologs of *WRI2*, along with *WRI1* and *WRI3*, during fruit development. While the role of WRI1 as a master regulator of oil biosynthesis is well-established, the function of WRI1 paralogs is poorly understood. Comprehensive and comparative *in silico* analyses of WRI1 paralogs from avocado (a basal angiosperm) with higher angiosperms *Arabidopsis* (dicot), maize (monocot) revealed distinct features. Predictive structural analyses of the WRI orthologs from these three species revealed the presence of AP2 domains and other highly conserved features, such as intrinsically disordered regions associated with predicted PEST motifs and phosphorylation sites. Additionally, avocado WRI proteins also contained distinct features that were absent in the nonfunctional *Arabidopsis* ortholog *At*WRI2. Through transient expression assays, we demonstrated that both avocado WRI1 and WRI2 are functional and drive TAG accumulation in *Nicotiana benthamiana* leaves. We predict that the unique features and activities of ancestral *PaWRI2* were likely lost in orthologous genes such as *AtWRI2* during evolution and speciation, leading to at least partial loss of function in some higher eudicots. This study provides us with new targets to enhance oil biosynthesis in plants.

## Introduction

WRINKLED1 (WRI1) belongs to the APETALA2/ethylene-responsive element binding protein (AP2/EREBP) class of transcription factors that play a master role in the developmental regulation of oil biosynthesis in plants (Cernac and Benning, 2004). The WRI1 transcription factor was first identified in *Arabidopsis*, where mutant seeds showed 80% reduction in triacylglycerol (TAG) content relative to wild-type (WT), 50% increase in sucrose levels, and a wrinkled seed coat phenotype. Subsequent studies revealed that WRI1 regulates a number of genes involved in controlling carbon allocation between sucrose and fatty acids in developing seeds (Focks and Benning, 1998). As such, *Arabidopsis wri1* mutant seeds were unable to efficiently convert carbon from sucrose and glucose into fatty acids during seed development. The genes regulated by WRI1 include *phosphoglycerate mutase, plastidial pyruvate kinase β-subunit 1 (PI-PKβ1), pyruvate dehydrogenase 1α (PDHE1α), biotin carboxyl carrier protein 2 (BCCP2), acyl carrier protein 1 (ACP1)*, and *keto-ACP synthase 1 (KAS1)* (Baud and Lepiniec, 2009; Maeo et al., 2009). Additionally, BIOTIN ATTACHMENT DOMAIN CONTAINING (BADC) proteins are conditional inhibitors of fatty acid biosynthesis; the genes encoding for *BADC1*, 2, and 3 were also positively regulated by WRI1 (Keereetaweep et al., 2018; Liu et al., 2019) Hence, fatty acid and storage lipid biosynthesis mediated through WRI1-induced genes is a complex and tightly regulated process.

DNA-binding studies and predictive structural analyses have shown that the regulation of glycolytic and fatty acid biosynthesis genes is achieved by the binding of AP2 domains in WRI1 with the AW-box, a 5′- upstream element ([CnTnG](n)_7_[CG]) in the promoter region of the target genes (Ruuska et al., 2002; Cernac and Benning, 2004; Baud et al., 2007, 2009; Maeo et al., 2009; Fukuda et al., 2013; Kuczynski et al., 2020). Specifically, in *Arabidopsis* WRI1 (*At*WRI1), two AP2 domains (73 and 62 amino acid residues, respectively) are present in the *N*-terminal domain. A small exon coding for the predicted residues “VYL” (valine-tyrosine-leucine) is a functionally important element in the first AP2 domain and is widely conserved among many WRI1 orthologs. Mutation in any of these three residues rendered complementation constructs insufficient to rescue the low seed oil phenotype of retransformed *wri1-1* plants (Ma et al., 2013). Furthermore, three intrinsically disordered regions (IDRs) were identified in *At*WRI1, with one located near the *N*-terminus, preceding the first AP2 domain, and the other two towards the *C*-terminal region, after the second AP2 domain (Ma et al., 2015). While part of the *C*-terminal region from 307 to 397 in *At*WRI1 is implicated in positive regulation of transcriptional activity (Ma et al., 2015), the occurrence of a PEST motif in nearby IDR3 likely acts as a recognition site for proteolytic degradation. The disordered state of IDR3 likely favors kinase access to key residues in this region and PEST residue phosphorylation serves as a signature that is recognized by key enzymes in the degradation pathway (Belizario et al., 2008). Since the PEST motif does not overlap with the transactivation domain (TAD), deletion or mutation of this motif increased the stability of WRI1 (Ma et al., 2015).

Since the discovery and characterization of WRI1 as a master regulator of fatty acid biosynthesis in seed tissues, its homologs have been identified in diverse plant species (Tang et al., 2019). In *Arabidopsis*, four paralogs of WRI1 (WRI1, WRI2, WRI3, and WRI4) were identified, all of which belong to the AP2/EREBP family of transcription factors. These genes, except *AtWRI2*, were also shown to play functional roles in fatty acid biosynthesis in various tissues (To et al., 2012). WRI1 is the dominant isoform that largely controls fatty acid metabolic gene expression during seed development and maturation. A basal level of transcriptional activity is however, maintained in *wri1* mutant seeds, suggesting a redundant role played by functional paralogs (Baud et al., 2009). Both *WRI3* and *WRI4* are highly expressed in floral tissue and stems, and thus contribute to fatty acid production for sustained cutin synthesis (To et al., 2012). Conservation of functional homology of WRI1 genes from other species such as *Zea may*s (Pouvreau et al., 2011), *Brassica napus* (Liu et al., 2010), and *Camelina sativa* (An et al., 2017) was shown by the complementation of *Arabidopsis wri1* mutants. In maize, there are two alternate splice forms of a *WRI1* gene (*ZmWRI1a* and *ZmWRI1b*); *ZmWRI1a* is strongly expressed in endosperm and clearly regulates kernel oil accumulation. The tissue-specific and temporal regulation of *ZmWRI1b* expression is different and suggestive of an ongoing evolutionary functional specialization of these two isoforms (Pouvreau et al., 2011). Transgenic expression of two *WRI1* isoforms from *B. napus* (*BnWRI1-1* and *BnWRI1-2*) increased the seed oil content by 20% in *Arabidopsis* seeds (Liu et al., 2010). Similarly, *Cs*WRI1 from *Camelina sativa* also partially restored the seed fatty acid levels in *Arabidopsis wri1* mutants; its transient expression also increased the TAG content in tobacco leaves (An et al., 2017). More recently, various transcriptome studies have extended the role of WRI1 in regulation of oil biosynthesis beyond seed tissues. The function of WRI1 in regulation of fatty acid synthesis in oil palm (*Elaeis guineensis*) mesocarp, which accumulates about 90% oil by dry weight, was established unequivocally (Bourgis et al., 2011; Ma et al., 2013). Furthermore, *Eg*WRI1 was able to rescue the low oil content in *Atwri1-1* seeds suggesting that WRI1 is responsible for oil accumulation in both seed and non-seed tissues (Ma et al., 2013) in some plant species. Additional studies have identified high expression of tissue-specific *WRI1* paralogs in diverse non-seed tissues such as Chinese tallow (Divi et al., 2016), potato, oat, and mesocarp of avocado (Kilaru et al., 2015; Rahman et al., 2016).

Avocado (*Persea americana*) is a basal angiosperm and occupies a useful evolutionary niche as one of the earliest lineages near the origin of flowering plants (Chanderbali et al., 2008). Avocado is also an ideal plant system to study oil biosynthesis for various reasons. The mesocarp of avocado accumulates 60–70% of oil by dry weight, of which, more than 80% is composed of nutritionally desirable oleic acid (Kikuta and Erickson, 1968; Kilaru et al., 2015). Additionally, transcriptome studies of developing mesocarp revealed that its oil accumulation is strongly correlated with the expression of fatty acid biosynthetic genes and *WRI* orthologs (Kilaru et al., 2015). Orthologs of all four members of the *AtWRI* gene family are present in avocado, referred to as putative *PaWRI1* to *PaWRI4*, and all but the *PaWRI4* are highly expressed in avocado mesocarp in correlation with oil accumulation. Avocado is unique in that it is the first plant system with confirmed concomitant expression of multiple *WRI1* paralogs during oil accumulation, which has not been observed previously. *PaWRI3* and *PaWRI4* are likely the functionally diverged products of a relatively recent gene duplication event, given that *PaWRI4* is not expressed in the presence of highly expressed *PaWRI3* (Kilaru et al., 2015). Since *AtWRI2* was unequivocally shown to lack any role in oil biosynthesis, high expression of its ortholog during non-seed oil accumulation in avocado was intriguing. Thus, we carried out comprehensive and comparative *in silico* analysis followed by transient expression studies to examine if *Pa*WRI2 was functional and associated with TAG accumulation.

## Materials and Methods

### Predictive Structural Analyses

Sequences of the WRI homologs for different species were retrieved from the NCBI database based on the BLASTP search using corresponding WRI-paralogs from *Arabidopsis* as query. Putative transcript sequences of *PaWRI1, 2, 3*, and *4* were obtained from the previously published avocado transcriptome data (Ibarra-Laclette et al., 2015; Kilaru et al., 2015; Rendón-Anaya et al., 2019). Full-length sequences were subsequently confirmed by cDNA synthesis and PCR amplification using gene specific primers, and sequencing, and deposited in the GenBank. The accession numbers for all the sequences used in this study are provided in the Data Availability Statement. Various bioinformatics tools were utilized for the subsequent *in silico* analyses (Supplementary Table 1).

The predicted protein sequence of each avocado gene was generated with the ExPASy translation tool. Multiple Sequence Alignment was done using the PRALINE online tool (Simossis and Heringa, 2003; Supplementary Table 1). For sequence comparison, all three avocado WRI paralogs were compared to their orthologs from *Arabidopsis* and maize, with protein sequences in FASTA format was used as inputs. BLOSUM62 exchange weights matrix was used, with gap penalties value 12 for open and 1 for extension. Homology-extended alignment was performed keeping all other parameters at the default values provided by the software. The outputs were obtained in a color-coded conservation scoring format where the conservation at each position of amino acid was denoted by a score from 0 to 10; 0 being the least-conserved position and 10 (indicated by asterisk) being the highest. The information about the positioning of AP2 domains in each WRI protein sequence was obtained from the NCBI database.

The amino acid composition profile of each protein was determined using COMPOSITION PROFILER (Vacic et al., 2007; Supplementary Table 1). Each of the protein sequences was used as a query sample against the SwissProt 51 dataset. The graphical output was obtained in a bar chart format composed of twenty data points for each amino acid arranged in increasing order of hydrophobicity (Eisenberg), where bar heights indicate enrichment (upward) or depletion (downward). The software also performs statistical analyses to evaluate whether the enrichment and depletion of amino acids were significant at a (α) of 0.05. Only the significantly enriched amino acids are represented in green and the depleted amino acids in blue.

Composition of each of the secondary structures, such as α-helix, β-strand, β-turn, and random coil structures, were predicted using the Self-Optimized Prediction Method with Alignment (SOPMA) secondary structure prediction online tool (Combet et al., 2000; Supplementary Table 1) with default parameters. Percentages for each of the predicted secondary structure components are denoted in the tables and the respective positions of each are expressed in graphical format.

The prediction of IDRs was conducted using the Predictors of Natural Disordered Regions (PONDR-VL3) online tool (Peng et al., 2005; Supplementary Table 1). The disordered regions in the full-length protein sequences were identified and provided as graphic format outputs. A score above the threshold value of 0.5 indicates a propensity of that stretch of protein to be a part of an IDR. Only the stretches of >30 amino acid residues showing disordered tendency are considered as likely IDRs in the protein tertiary structure.

Kinase phosphorylation potential of each Ser (S), Thr (T), or Tyr (Y) residue was evaluated using the NetPhos 3.1 online tool (Blom et al., 1999). The likelihood threshold values were considered at 0.5. The positions of putative PEST motifs were predicted using the epestfind online tool (Supplementary Table 1).

### Statistical Analysis

The transient expression assays were performed in triplicate and data were expressed as their mean value with standard deviation (SD). To test for significance amongst the dataset, one-way analysis of variance (ANOVA) followed by Tukey’s post-test was performed using Minitab software (version 18) at *p*-level of 0.05 (*p* < 0.05).

### cDNA Synthesis and Cloning

Total RNA extract from avocado mesocarp was available from the previous study (Kilaru et al., 2015). From the total RNA, cDNA was synthesized using oligo dT primer and superscript reverse transcriptase (Promega). Full-length coding sequences of putative *PaWRI1* and *PaWRI2* were amplified by PCR using specific primers (*PaWRI1-F*: 5′-GCTCCC**ATG**GACACATCTTCTCCCCTCTCCAATT-3′ and *PaWRI1-R*: 5′-CTATCCGCGGCTAAGAACATATGCTGATGG GAAGCGGAT-3′ for *PaWRI1*; *PaWRI2-F* : 5′-GCTCCC **ATG**GCTTCTTCTCCTTCGTCGTCG-3′ and *PaWRI2-R*: 5′-GCGGTCCGCGGTTATTCTTGACGGAAGAAAGTATATG TTG-3′ for *PaWRI2*) containing *Nco*I and *Sac*II restriction sites (underlined) in their forward and reverse primers, respectively. Initiator methionine codons in the forward primers are shown in bold. The PCR products were digested with appropriate restriction endonucleases, and cloned into the corresponding sites in entry vector pK34 with a dual CaMV35S promoter and terminator (Shockey et al., 2015) using T4 DNA ligase (New England Biolabs). Ligation reactions were chemically transformed into Top10 *E. coli* competent cells, grown on solid media containing ampicillin, and insert-positive colonies were identified by colony PCR. Insert sequence accuracy was confirmed by sequencing. For expression in tobacco leaves, *Asc*I fragments representing the promoter:gene:terminator cassettes from the K34-based entry plasmids were cloned into the binary expression vector pB110 (Shockey et al., 2015), followed by another round of *E. coli* transformation and colony selection, this time using kanamycin as the selective agent. The viral silencing suppressor protein gene P19 (Wood et al., 2009) was also cloned into pB110 behind the CaMV35S promoter using this strategy, and was later used for co-infiltration into *N. benthamiana* leaves.

### Transient Expression

Wild type tobacco (*Nicotiana benthamiana*) was used for all transient expression experiments. All plants were grown in a growth chamber (Percival Scientific, Perry, IA, United States) under long day conditions (15 h L/9 h D) at 24°C and 60% relative humidity. Six weeks old plants were used for transient expression. For plant transformation, 100 μL of *Agrobacterium* LBA4404 competent cells were gently mixed with 100-1000 ng of binary plasmid DNA expressing the gene of interest, kept on ice for 5 min, then immediately transferred into liquid nitrogen. Subsequently, the flash-frozen mixture was incubated for 5 min at 37°C in a water bath and then transferred into 1 mL of LB media and incubated for 2-4 h at 28°C. After the incubation period, the bacterial cells were pelleted by gentle centrifugation and resuspended in 100 μl LB and immediately plated onto LB agar containing kanamycin (50 μg/mL) and rifampicin (50 μg/mL). Positive colonies were used for overnight culture in LB liquid media containing appropriate antibiotics at 28°C. The overnight culture was further supplemented with 100 μM acetosyringone and allowed to grow for additional 2 h. After incubation, the solution was centrifuged at 400 × *g* for 5 min at room temperature and the pellet was resuspended in infiltration buffer (5 mM MgSO_4_, pH 5.7, 5 mM methyl ethanesulfonate, and 100 mM acetosyringone). Finally, *Agrobacterium* constructs with an O.D. of 0.3 at 600 nm were prepared and infiltrated into *N. benthamiana* leaves. After infiltration, plants were maintained under ambient conditions in the growth chamber, to avoid any external stressors. Plants were allowed to grow for 6-7 days to express the recombinant protein. Subsequently, inoculated areas of leaves were harvested and either processed immediately or stored at −80°C for further analyses.

### Nile Red Staining

After *Agrobacterium* infiltration, *N. benthamiana* plant leaf discs were collected and fixed with paraformaldehyde in 1X phosphate-buffered saline (PBS). Nile Red (in dimethyl sulfoxide) was diluted in 1X PBS (final concentration 4 μg/mL) was used for lipid droplet staining; leaf discs were immediately observed using a Leica TCS SP8 confocal fluorescence microscope. The excitation and emission wavelength for Nile Red staining were 488 nm and 560-620 nm, respectively. Images were acquired as a z-stack of 10 optical sections. The dimensions of each panel were 161.7 × 161.7 μm and the total number of LDs per view were quantified using ImageJ software the count was normalized to LD/mm^2^. Images were obtained from three biological replicates.

### Extraction of Plant Lipids

Other infiltrated *N. benthamiana* leaf discs were collected at 6-7 days after *Agrobacterium* infiltration and used for total lipid extraction. Lipids were extracted using the hexane:isopropanol method (Hara and Radin, 1978). Briefly, 200 mg of plant tissue (fresh weight, FW) was ground in liquid nitrogen and 2 ml hot isopropanol was added, then heated to 70°C for 30 min in water to inactivate any internal lipase activity. After cooling to room temperature, one mL of chloroform and 250 μL water were added to achieve a final ratio of 2 mL isopropanol: 1 mL chloroform: 0.45 ml water. The samples were stored O/N at 4°C. The next day, the supernatant was collected from the sample after vortexing and centrifugation for 5 min at 5000 × *g*. One mL of chloroform and 2 mL of 1M KCl were added to the sample to achieve phase separation. After centrifugation, the upper aqueous phase and interphase were aspirated by using a Pasteur pipette. The sample was then washed twice with 2 mL of 1M KCl and the organic phase was collected and dried under nitrogen gas. Dried lipid samples were reconstituted in ∼1 mL of chloroform and transferred into a pre-weighed glass vial. After evaporating the chloroform in nitrogen gas, each glass vial was weighed again to obtain the total lipid weights. Before homogenization, an appropriate amount of heptadecanoic acid, 17:0 standard (100 μg; Sigma-Aldrich) was added to the plant tissue.

### Fatty Acid Analysis by Gas Chromatography-Flame Ionization Detection (GC-FID)

Total lipids extracted from the infiltrated leaves were esterified to determine fatty acid composition. To perform fatty acid methyl esterification (FAME), HCl methanolic acid (1 N) was added to the samples and the mixture was heated for 2 h at 85°C in a water bath. After cooling to room temperature, 1 mL KCl and 1 mL hexane were added for phase separation. The hexane organic phase containing FAMEs was collected and dried under nitrogen gas at 40°C. Each FAME sample was resuspended in 1 μL of hexane and analyzed by gas chromatography coupled with a flame ionization detector (GC-FID, Varian). A capillary column (DB-23; 30 m × 0.32 mm I.D., 0.25 μm) with helium as carrier gas (flow rate of 1.5 mL/min) was used. The injection temperature was set at 150°C, which was ramped after 3 min to 240°C at 6°C/min; the detection temperature was 300°C. The retention time for each fatty was determined based on comparison to the Supelco FAME mix standards, and quantified relative to the C17:0 internal standards. Each sample was analyzed in triplicate.

## Results and Discussion

### Primary Sequence Features of WRI Paralogs in Avocado Differ From Their Dicot and Monocot Orthologs

**FIGURE 1:**
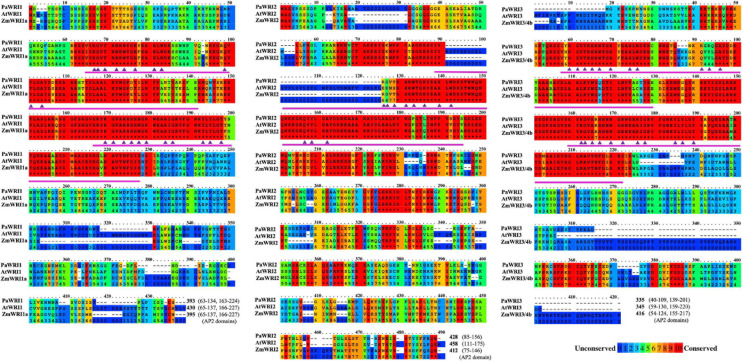
Primary sequence features of WRI homologs. Multiple sequence alignment of primary sequences of WRI1, 2, and 3 from *P. americana* (Pa), *A. thaliana* (At), and *Z. mays* (Zm) in a color-coded format; red and blue colors represent the most (10, indicated by asterisk) and least (0) conserved regions, respectively, on a scale of 0 to 10. Position of the AP2 domains and sequence length are indicated at the end of the alignment. The amino acid residues in the AP2 domain important for DNA interaction are represented by (▲).

To identify distinct structural characteristics among the WRI orthologs, comprehensive *in silico* analyses were conducted. Initially, phylogenetic analyses of WRI orthologs from select basal angiosperms (avocado and *Amborella*), monocots (rice, maize, and oil palm) and dicots (*Arabidopsis*, rapeseed, castor, grape, and poplar) was carried out (Supplementary Figure 1). These analyses revealed that WRI2-orthologs evolved independently and earlier than their corresponding paralogs in the respective plant species (Supplementary Figure 1). Further comprehensive analyses of structural features of *Pa*WRI2, relative to its paralogs and orthologs specifically from maize and arabidopsis were determined by protein sequence comparison. Among the three species, the WRI1 protein sequences shared an overall identity of 50%, while WRI2, WRI3, and WRI4 showed 45, 57, and 59% identity, respectively (Figure 1). Additionally, phylogenetic analysis showed that avocado WRI3 and WRI4 share > 70% identity and are likely the result of a gene duplication event (Supplementary Figure 1). Duplicated genes may retain functional redundancy, but in some cases attain specialized temporal- or tissue-specific roles. *PaWRI3* transcripts were abundant and *PaWRI4* was poorly expressed in avocado mesocarp (Kilaru et al., 2015), suggestive of a larger role for *PaWRI3* in mesocarp lipid metabolism. Whether the functional role of WRI2 orthologs in oil biosynthesis is conserved in any of the plant species has not been elucidated, except for *At*WRI2, where it was shown to be non-functional based on its inability to complement *Atwri1-1* mutant (To et al., 2012). As such, only WRI1, WRI2, and WRI3 orthologs were included in the subsequent *in silico* comparisons.

Primary sequence comparisons revealed that avocado WRI1 and WRI3 proteins were generally shorter than their corresponding orthologs in *Arabidopsis* and maize (Figure 1) and also to some of other monocot and dicot orthologs examined (Supplementary Figure 2). Specifically, *Pa*WRI1 is shorter than *At*WRI1 and *Zm*WRI1a by 37 and 2 amino acids, respectively. *Pa*WRI3 is only 10 amino acids shorter than *At*WRI3, but 81 amino acid shorter than *Zm*WRI3/4b. *Pa*WRI2 is 30 amino acids shorter than *At*WRI2 and 16 amino acids longer than *Zm*WRI2. Interestingly, the relative position of the regions that contributed to shorter avocado WRI sequence among its paralogs varied. In the case of WRI1, both avocado and maize lacked several amino acids downstream of the second AP2 domain that are present in *At*WRI1, while for WRI2, several amino acids in both *N*-terminal and *C*-terminal regions were absent. However, for WRI3, *Pa*WRI3 and *At*WRI3 lacked several *N*- and *C*-terminal amino acids that are present in *Zm*WRI3/4b. More than 45% of the shared identity is found in the *N*-terminal AP2 domains, which is a distinct feature of the APETALA protein family. As a result, the *N*-terminal region of WRI1 orthologs in different plant species is highly conserved, whereas the *C*-terminal region that harbors the TAD is variable (Kong et al., 2019; Tang et al., 2019; Fei et al., 2020).

Among the two highly conserved *N*-terminal AP2 domains, the first domain is typically more conserved than the second (Cernac and Benning, 2004), although both were similarly, conserved across the orthologs of the three species examined in detail here. Also, while both AP2 domains are present in the WRI1, WRI3, and WRI4 orthologs, the WRI2s, including *Pa*WRI2, lacked the second AP2 domain (Figure 1 and Supplementary Figure 2). Moreover, unlike *Pa*WRI2 or *Zm*WRI2, the single AP2 domain present in *At*WRI2 was interrupted by a stretch of 35 additional amino acids (Figure 1 and Supplementary Figure 2). Plant AINTEGUMENTA transcription factors are able to bind DNA promoters despite having only a single AP2 domain (Krizek, 2003), but yeast-one hybrid studies have shown that *At*WRI2 was unable to bind to the promoter of the *BCCP2* gene (To et al., 2012), likely due to the interruption present in the AP2 domain. The need for an intact AP2 domain to bind DNA promoter motifs might explain the lack of function for *At*WRI2; whether this function exists in *Zm*WRI2 remains unexplored.

The WRI AP2 domains interact with the DNA elements called the “AW-box” in target gene promoters (Cernac and Benning, 2004). While the identity of the protein residues that bind to the AW element is not yet known, based on the sequence and structural similarity to the GCC-box binding domain (GBD) of *At*ERF1 (1GCC; PDB database), 11 amino acid residues in the AP2 domain were predicted to interact with the target promoters (Okamuro et al., 1997; Allen et al., 1998). While these 11 amino acid residues are conserved among the WRI1 orthologs, *At*WRI2 showed mutation in the first three residues (R→E, G→E, & T→S) and *Pa*WRI3 had mutations in two residues (Q→K, Y→R) (Figure 1 and Supplementary Figure 2). The evolution of these three residues could also contribute to the loss of function in *At*WRI2; DNA binding activity in *Pa*WRI3 is yet to be explored.

Although the larger *N*-terminal region is highly conserved overall, the sequence upstream of the AP2 domains is variable among the orthologs. Particularly, the presence of repeat regions of serine residues in *Pa*WRI1 and glycine in *Pa*WRI2 is similar to their respective orthologs in *Arabidopsis* but not in maize. The serine-rich region in the *N*-terminus is thought to have gradually evolved among vascular plants (Tang et al., 2019), which may play a role in functional regulation and subcellular localization (Casal et al., 2002; Ma et al., 2015), whereas the role of glycine repeats in *Pa*WRI2 is not known. The micro-exon encoding the “VYL” motif in the first AP2 domain is essential for *At*WRI1 function (Ma et al., 2013). This domain is present in all WRI proteins here, except for *Pa*WRI3 and *Pa*WRI4 (Figure 1). Previous studies showed that site-directed mutagenesis of the amino acids “VYL” in *At*WRI1 failed to complement *Atwri1-1* mutant but the WRI1 isoform in castor bean (*Rc*WRI1-B) and rice (*Os*WRI1-1) were functionally active despite lacking the motif (Ma et al., 2013; Ji et al., 2018; Mano et al., 2019). Thus, the mechanistic role of the “VYL” motif is not well-understood, and the functional importance appears to be species-dependent. High *PaWRI3* expression during oil accumulation suggests that this protein might be functional, even in the absence of the motif. Additionally, two KIN10-mediated phosphorylation target residues (T70 and S166) identified in *At*WRI1 are conserved in all the avocado paralogs, but S166 is absent in both *At*WRI2 and *Zm*WRI2 (Supplementary Figure 3). Phosphorylation of these two residues by KIN10 triggers WRI1 degradation as a response to elevated sugar levels in the cell (Zhai et al., 2017). This suggests a possible *Pa*WRI2-mediated, sugar-dependent regulation of avocado oil biosynthesis (that is likely aided by *Pa*WRI1 and *Pa*WRI3 as well), while such a mechanism is lost for WRI2 orthologs in at least some higher plants.

Additionally, other interesting protein structural variations were also observed. In general, the *C*-termini share very little sequence similarity. An acidic residue-rich region that overlaps with the putative TAD in *Arabidopsis* WRI1 was identified in monocots and other dicots (Ma et al., 2015; Tang et al., 2019). However, the TAD in *At*WRI1 (307-397) is not conserved in other orthologs and about twenty six amino acid deletions were identified in that region of *Pa*WRI1 (Figure 1). Among the three WRIs in avocado, *Pa*WRI2 has the longest *C*-terminal region (sequences downstream of AP2 domain; 272 residues, compared to 169 and 134 in *Pa*WRI1 and *Pa*WRI3, respectively) although the length variation is commonly observed in other plant species (Tang et al., 2019). Also, both *Pa*WRI3 and *At*WRI3 lack several amino acids in their *C*-terminal domains, compared to their maize ortholog with an extended *C*-terminus (Figure 1).

### Avocado WRI Sequences Include the Signature of a Structurally Disordered Protein Fold

Amino acid composition biases are important for protein function and are prominent among eukaryotic transcription factors. Particularly, enrichment with hydrophilic or depletion of hydrophobic amino acids leads to generation of a disordered protein fold that is associated with proteostasis (Dyson and Wright, 2005; Gao and Xu, 2012). The locally present unstructured regions due to amino acid composition biases are referred to as intrinsically disordered regions (IDRs) and the proteins as intrinsically disordered proteins (IDPs; Liu et al., 2006). The disordered value of *At*WRI1 was ∼53% due to composition bias (Ma et al., 2015); similar analysis of other WRI homologs also revealed enriched polar amino acids and depleted hydrophobic amino acids (Supplementary Figure 4). Sequence alignment analysis has already indicated a great degree of dissimilarity in *N*- and *C*-terminal regions flanking the AP2 domains. To investigate compositional preference and to search for regions of significant enrichment, we used the SwissProt 51 database as a standard reference. The data showed that serine (S) is enriched in all the homologs, except *Zm*WRI3/4b. Hydrophobic amino acids like Ile (I), Val (V), and Leu (L) were significantly depleted in most of the WRI homologs (Supplementary Figure 4). For example, L and V were depleted in *Pa*WRI1 and *Pa*WRI2, respectively, while all the three residues (I, V, and L) were depleted in *Pa*WRI3. However, unlike the other orthologs, the polar amino acid Lys (K) was significantly depleted only in *Pa*WRI1 (Supplementary Figure 4). The tendency for enrichment of polar amino acids or depletion of hydrophobic amino acids was highest in *At*WRI1 and lowest in *Pa*WRI1, while the contrary was true for WRI3 orthologs. Among the WRI2 orthologs, however, the propensity was higher for *Zm*WRI2 followed by *Pa*WRI2 and then *At*WRI2. Overall, the degree of positive S bias and depletion of non-polar amino acids like I, V, and L suggests that WRI transcription factors in avocado have a characteristic feature of IDPs.

**FIGURE 2:**
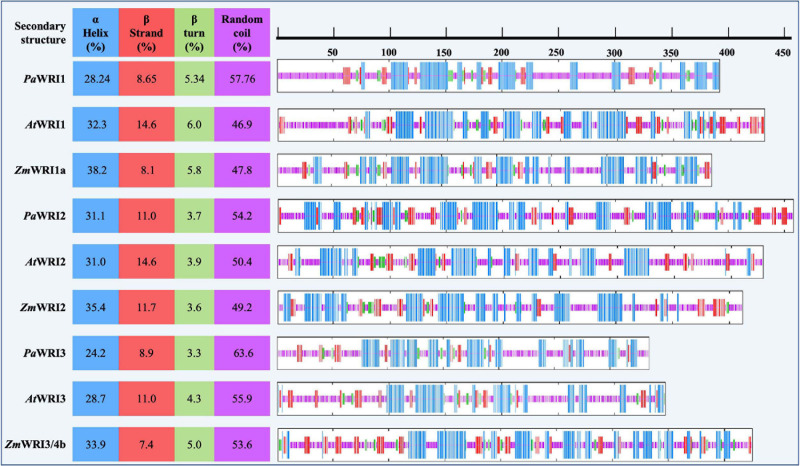
Proportion of the predicted secondary structures in WRI homologs. Position of each of the secondary structures α helix (blue), β strand (red), β turn (green), and random coil (pink) among all the WRI1 homologs are schematically represented on the right. The color-coded regions represent the position of the corresponding secondary structures. Proportion of each of the secondary structures is represented as a percentage value on the left, for each homolog.

A disorder-promoting amino acid composition among the WRI homologs suggests that achieving secondary structure folds would be difficult. A hydrophobic core is important for nucleation of protein folding in an aqueous environment; depletion or low proportions of hydrophobic amino acids will typically lead to random coil (Eisenberg et al., 1986). Unstructured folds or random coil regions are common among eukaryotic proteins including transcription factors (Uversky and Dunker, 2010). Such features enable proteins to adopt different conformations to interact with multiple target partners and other coregulatory proteins (Khan and Kumar, 2009). The composition analyses of WRI homologs were used to predict the degree of secondary structure (α-helix, β-sheet, β- turn, and random coil) for each protein. In all the homologs, random coil structure was the highest in proportion, followed by α-helix, β-strand, and β-turn (Figure 2). All the avocado WRI paralogs have > 50% random coil region, which is greater than in their respective orthologs in *Arabidopsis* and maize. The random coil regions are mostly present in the *N*- and *C*-terminal regions, while the AP2 domain regions contain abundant α- helix and β-sheet content and a low proportion of random coils (Figure 2). The crystal structure of the GCC-box binding domain (similar to AP2 domain) of EREBP protein also has the same feature, where the β-sheets were particularly responsible for DNA binding (Allen et al., 1998). Although the 3D structure of the AP2 domains is yet to be resolved, based on the sequence homology, it is expected that their promoter binding ability is also achieved in a similar manner. Additionally, extensive random coil structure flanking the WRI AP2 domains can also contribute to effective binding to different promoters. Such functionality is evident from the demonstrated ability of *At*WRI1 to bind to the promoter regions of various fatty acid synthesis genes (Baud and Lepiniec, 2009; Maeo et al., 2009). Overall, it can be inferred that a high proportion of random coil structure associated with WRI homologs, including those from avocado, contributes to high conformational flexibility to interact with their target genes resulting in a complex network of WRI-mediated regulation of oil biosynthesis. These findings support the possibility that *Pa*WRI1, *Pa*WRI2, and *Pa*WRI3 have retained relatively more flexible and dynamic structures that allow for greater conformational promiscuity to interact with numerous target proteins.

### Avocado WRI2 Lacks the *C*-Terminal IDR3 Region

Intrinsically disordered regions in the protein structure can prevent the formation of a hydrophobic core and result in improper folding (Uversky et al., 2000; Romero et al., 2001). In all three WRI homologs, in addition to the local random coil structures, amino acid bias also contributed to IDRs. The propensity for disorderliness was about two-fold higher among WRI homologs analyzed, compared to the mean value of 23% observed in the general *Arabidopsis* proteome (Oldfield et al., 2005). The overall disorder percentage among WRI1 orthologs was as follows: *Zm*WRI1a > *At*WRI1 > *Pa*WRI1, whereas for WRI2 orthologs, *Pa*WRI2 > *Zm*WRI2 > *At*WRI2 and for WRI3, *Zm*WRI3/4b > *Pa*WRI3 > *At*WRI3 (Table 1). While a higher percentage of disorderliness promotes flexibility and protein interaction, accessibility to proteases and the likelihood of protein degradation also increases. So, the relatively higher disordered values associated with both *Pa*WRI2 and *Pa*WRI3 and lower values with *Pa*WRI1 might reflect a complex homeostasis, in which one protein could compensate for the degradation of the other to achieve sustained fatty acid biosynthesis during the prolonged period of TAG accumulation in avocado mesocarp.

**TABLE 1:** Details of the disorder value and the position of various intrinsically disordered regions (IDRs) and PEST motifs in WRI homologs.

**Proteins**	**% Disorder**	**IDR1**	**IDRla**	**IDR2**	**IDR3**	**PEST motifs**
PaWRIl	51.4	1-67	*	226-273	314-375	1-35,228-247
AtWRIl	52.5	1-71	*	249-313	368-430	3.34, 396-430
ZmWRIla	56.7	1-67	135-175	238-313	356-395	6-32,232-273,273-319
PaWRI2	54.2	1-85	171-201	266-381	*	*
AtWRI2	45.4	1-57	*	278-377	426-458	*
ZmWRI2	48.0	1-74	*	247-319	362-412	*
PaWRB	55.8	1-53	115-147	215-315	*	206-242, 277-295
AtWRB	52.7	1-70	137-168	236-314	*	*
ZmWRB/4b	57.9	1-63	131-164	246-362	*	302-344

*Indicates the absence of the corresponding IDR or PEST motif.

**FIGURE 3:**
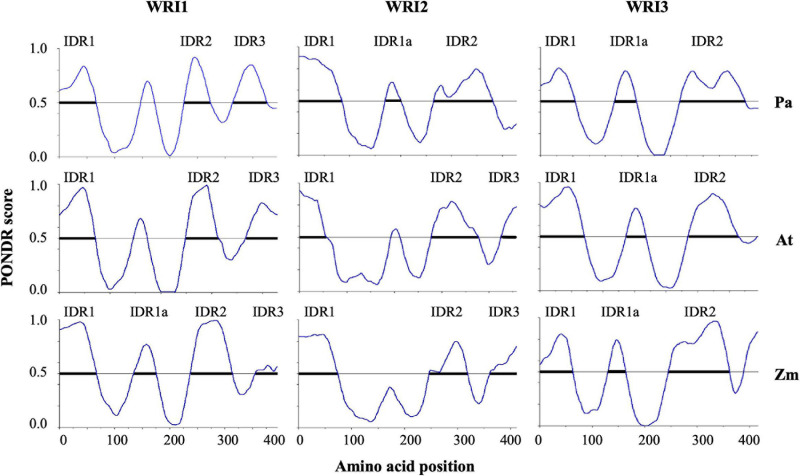
Graphical representation of the predicted IDRs in the WRI homologs. The predicted IDRs in all the WRI homologs are graphically plotted with the amino acid position on the *x*-axis and the PONDR score on the *y*-axis. Regions showing PONDR score > 0.5 are considered as IDRs.

The percentage disorder observed was reflected in the number of IDRs identified in each homolog. The protein sequences of *Pa*WRI1, *At*WRI1, and *Zm*WRI1 were predicted to contain three, three and four IDRs, respectively; all WRI2 and WRI3 proteins showed the presence of three IDRs (Figure 3). For further comparisons, the identified IDRs were named based on their position, similar to previously identified IDRs in *At*WRI1, i.e., IDR1, IDR2, and IDR3. While IDR1 and IDR2 were unanimously predicted in all homologs, avocado WRI2 and WRI3 paralogs lacked the IDR3 in their *C*-terminal region. An additional short IDR (30-40 aa), which we referred to as IDR1a, was predicted in the linker region connecting the two AP2 domains (*Zm*WRI1a and all the three WRI3) and following the single AP2 domain in *Pa*WRI2 proteins (Figure 3). Interestingly, the size of the IDRs was quite variable among the homologs (Table 1). Long IDRs (>30 aa) are common, comprising ∼33% of the eukaryotic proteome and affect transcription and translational activity, post-translational modifications, transportation and signal transduction activity, and also stability and degradation (Ward et al., 2004; Dyson and Wright, 2005; Van Der Lee et al., 2014). Shorter IDRs in the linker regions typically offer conformation flexibility that is needed for DNA-binding proteins (Tompa, 2005; Van Der Lee et al., 2014; Davey, 2019). The shorter IDR1a in the linker regions of some WRI orthologs, as well as the other longer IDRs around the AP2 domains are likely to provide conformational flexibility for an effective interaction with the target promoters while IDRs in the C-terminus might be associated with protein stability.

Disordered *N*- and *C*-terminal regions are common in DNA-binding proteins; *C*-terminal IDR is especially prevalent in transcription factors (Van Der Lee et al., 2014). Previously characterized *At*WRI1 IDR3 was shown to have a functional role in transactivation, although a three-fold increase in expression upon the truncation of IRD3 suggested that its presence likely also serves as a target for protein degradation pathways (Ma et al., 2015). IDR3 is absent in *Pa*WRI2 although the other analyzed WRI2 orthologs have an IDR in their *C*-terminus (Figure 3 and Supplementary Figure 5). However, the *Pa*WRI2 contains relatively long IDR1 and IDR2 (85 and 116 residues) compared to the respective orthologs in *Arabidopsis* and maize (57 aa and 100 aa, and 74 and 73 aa, respectively). These IDRs likely provide the necessary degree of transactivation capacity, while such function was lost in *At*WRI2. The functional role of *Zm*WRI2 remains to be examined. Similarly, IDR3 is absent in yellow nutsedge (*Cyperus esculentus*) WRI1 (*Ce*WRI1), although it retained its functional role in oil biosynthesis (Grimberg et al., 2015) and showed autoregulatory effect on the *AtWRI1* promoter (Snell et al., 2019). Hence, it is possible that *Pa*WRI2 is still able to activate the target genes in a similar manner to the transactivation mechanism shown by *Ce*WRI1. On the other hand, long IDRs are also preferential targets for post-translational modification and protein binding that protect it from proteolytic cleavage (Dyson and Wright, 2005). The stability of *At*WRI1 is enhanced upon interaction with either BTB/POZMATH1 (BPM1) or 14-3-3 proteins (Chen et al., 2013; Ma et al., 2016), although the complete interaction network is currently unknown. Hence, it is possible that the difference in functionality between *Pa*WRI2 and *At*WRI2 could be the result of distinct post-translational modifications and/or protein interactions.

### The *C*-Terminal Region of Avocado WRI2 Lacks a PEST Motif

Among the WRI homologs examined, the *C*-terminal region downstream of the AP2 domain is highly variable (Figure 1). Nevertheless, this region does harbor some conserved features, without any particular trend in amino acid properties that are likely responsible for the regulation of the protein. Specifically, a region of the protein sequence that is enriched in proline (P), glutamic acid (E), serine (S), and threonine (T), referred to as PEST motifs occur among WRI homologs and were mostly associated with the IDRs (Ma et al., 2015). These PEST motifs are typically involved in proteasome-mediated protein turnover (García-Alai et al., 2006; Belizario et al., 2008). They are abundant among many key metabolic enzymes, transcription factors, protein kinases, and phosphatases in the cell, and function as a tool to maintain cellular homeostasis (Rechsteiner and Rogers, 1996). PEST-containing proteins make up a prevalent portion (∼25%) of the eukaryotic proteome and are enriched with disorder-promoting amino acids. The PEST motif associated with IDRs promotes proteolysis through ubiquitin-proteasome degradation or calpain cleavage (Gregory and Hann, 2000; Bordone and Campbell, 2002; Singh et al., 2006).

**FIGURE 4:**
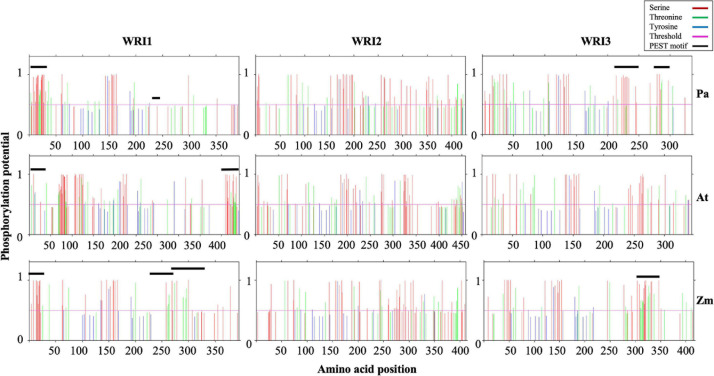
Schematic presentation of potential phosphorylation sites in the WRI homologs. Predicted phosphorylation potential for S (Red), T (green), and Y (blue) residue and their amino acid position are shown in the plots. The residues with potential higher than threshold value of 0.5 have higher probability of being phosphorylated. Residues that correspond to PEST motifs are also indicated by a black line.

While all three WRI1 orthologs contained one PEST motif in their *N*-termini, *At*WRI1 and *Pa*WRI1 had one each, and *Zm*WRI1a had two, PEST motifs predicted in their respective *C*-terminal IDRs (Figure 4 and Table 1). However, the PEST motif in the *C*-terminal region of *Pa*WRI1 has no potential phosphorylation site (Figure 4), and thus is an unlikely protease target. Interestingly, all WRI2 orthologs (including *Pa*WRI2) and *At*WRI3 lacked PEST motifs. Maize and avocado WRI3 orthologs, although lacking *N*-terminal PEST motifs, contained one and two each, respectively, embedded in their *C*-terminal IDRs (Figure 4 and Table 1). The association of PEST motifs with the IDR increases surface accessibility and favors it as a target for degradation signal attachment and phosphorylation (Singh et al., 2006). Additionally, while both *N*- and *C*-terminal PEST motifs are involved in proteolysis, a preference for *C*-terminal PEST motifs is noted in several proteins (Rechsteiner and Rogers, 1996; Singh et al., 2006). Among the WRI homologs, most PEST motifs were associated with the *C*-terminus and likely play a role in transcription factor stability control.

The PEST motif associated with *At*WRI1 IDR3 participates in phosphorylation-dependent protein degradation but was not critical for transactivation (Ma et al., 2015). Furthermore, transient expression of truncated *At*WRI1 (1-397) in *N. benthamiana* leaves increased oil accumulation suggesting that the PEST motif plays a role in protein accumulation without affecting basic function (Ma et al., 2015). Hence, the absence of a *C*-terminal IDR-associated PEST motif in *Pa*WRI2 might contribute to its increased stability and sustained activity, possibly contributing to high oil accumulation in avocado mesocarp. Although *PaWRI3* transcript levels were high in avocado mesocarp during the period of oil accumulation, any effects of PEST motifs on transcript accumulation are currently unknown (Kilaru et al., 2015). The role of *N*-terminal PEST motifs in WRI homologs also has not been studied but is predicted to maintain homeostasis and protein interaction. Overall, our analyses suggest that lack of a PEST motif in the *C*-terminal region of *Pa*WRI2, versus its presence in this region of *Pa*WRI1 and *Pa*WRI3, contributes to the mediation of the complex regulatory mechanism of homeostasis in avocado mesocarp tissue.

### Both *Pa*WRI1 and *Pa*WRI2 Contain Phosphorylation Sites in the *C*-Terminal Region

**FIGURE 5:**
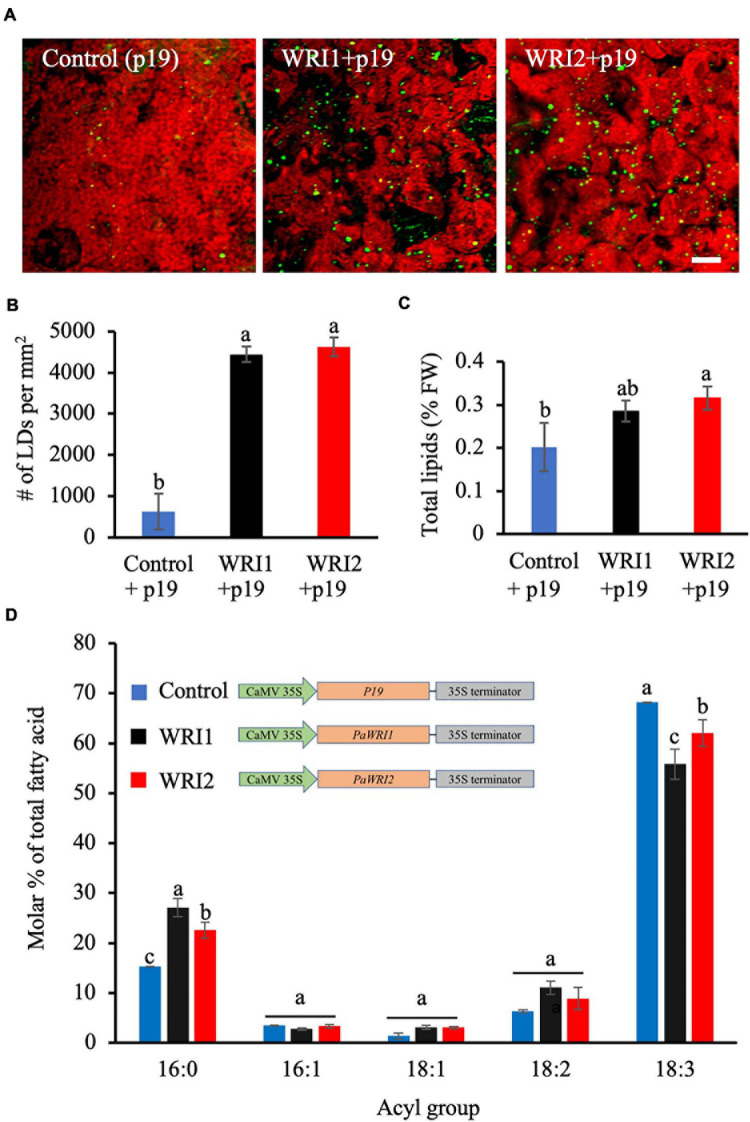
Lipid content and fatty acid composition of *N. benthamiana* leaves expressing *PaWRI1* and *PaWRI2*. **(A)** Confocal images of accumulated lipid droplets (LDs) stained with Nile Red (green) in *N. benthamiana* leaves infected with *Agrobacterium* expressing control (*p19* alone), *PaWRI1* (*+p19*), and *PaWRI2* (+*p19)* constructs. Scale bar corresponds to 20 μm. **(B)** Quantification of accumulated LDs per unit surface area of the leaf tissue. **(C)** Quantification of total lipid (TL) content. **(D)** fatty acid profile of total lipids extracted from the infiltrated leaves. Data represent mean ± sd (*n* = 3; *p* < 0.05).

Phosphorylation is among the major categories of post-translational modifications that control protein function. Phosphosites present in the long disordered regions in IDPs are preferentially phosphorylated by cellular kinases (Koike et al., 2020). To investigate the possible targets in our WRI protein set, we evaluated each of the putative residues Ser (S), Thr (T), and Tyr (Y) for phosphorylation modification potential using NetPhos 3.1 (Blom et al., 1999). The extensive S residues were most often identified as kinase target sites, followed by T and Y. Most of the identified phosphorylation sites overlapped with the predicted PEST motifs (Figure 5). Previous analysis of *At*WRI1 identified four S residues in the IDR3-PEST

motif as potential phosphorylation targets, and mutation of all these residues to Ala (A) (*At*WRI1^4SA^) resulted in increased stability and increased TAG accumulation in transiently expressed *N. benthamiana* leaves (Ma et al., 2015). Possibly, the Ser (S) residues are important and/or sufficient for the PEST motif recognition and phosphorylation by target enzymes to upregulate protein degradation. Since both *Pa*WRI1 and *Pa*WRI2 lack PEST motifs in their *C*-terminal ends, those regions did not show an enrichment of phosphorylation sites (Figure 4); these patterns thus differ significantly from those of *At*WRI1 and *At*WRI2. However, *Pa*WRI2 was enriched with potential phosphorylation sites after amino acid position 350, which were absent in *At*WRI2. Also, the region between the two AP2 domains in WRI1 and WRI3 orthologs and the region downstream of the AP2 domain in WRI2 orthologs were enriched with phosphorylation targets. Additionally, two KIN10-mediated phosphorylation target residues (T70 and S166, present in the first and second AP2 domains of *At*WRI1) were also conserved in all WRI homologs except *At*WRI2 and *Zm*WRI2 (Zhai et al., 2017). Although a large fraction of the identified phosphorylation sites remains unexplored, the high proportion of random coil structure and abundant IDR regions associated with each of the WRI proteins, including the avocado homologs, likely favor cellular protein kinase access, resulting in structural and/or functional modulation. Hence, phosphorylation, whether associated with residues in PEST domains or elsewhere, is also likely to play an important role in maintaining homeostasis of WRI paralogs in avocado mesocarp.

### Distinct Features of *Pa*WRI2 Are Likely Associated With Its Function

Previous studies reported the differentiation of avocado WRI2 from the other gene family members, likely due to an ancient gene duplication event. The loss of function in WRI2 from more recently evolved plant families such as *Arabidopsis*, suggests loss of selective pressure and enzyme function during evolution (Kilaru et al., 2015). We identified characteristic features of avocado WRI2 that might explain the retention of its functional role, through *in silico* analyses. All the WRI2 orthologs share only one AP2 domain, suggesting that it is functionally very important. Previous studies (Ma et al., 2013) suggested that the presence of a micro-exon, encoding the three residues “VYL,” is present in WRI1 orthologs from diverse plant species and is highly correlated with WRI protein functionality. *PaWRI2* retains this exon, while *AtWRI2* does not (Figure 1), suggesting that this element contributes to *Pa*WRI2 protein function. Another unique difference between *At*WRI2 and *Pa*WRI2 is the absence of IDR3 but presence of IDR1a in *Pa*WRI2 (Figure 3). Previous studies have reported that post-translational modifications in the *C*-terminal IDRs affect the stability of WRI1 proteins, for example, phosphorylation of the IDR3 PEST motif in *At*WRI1 enhances protein degradation (Ma et al., 2015). As such, IDR3 in *At*WRI2 might negatively affect its conformational flexibility and hence contribute to its loss of function. Conversely, in *Pa*WRI2, the presence of IDR1a adjacent to the single AP2 domain (Figure 3) might contribute to productive interaction with target promoters. Furthermore, retention of function in *Pa*WRI2

might be attained due to the presence of only one *C*-terminal IDR that does not contain PEST motifs (Figure 3 and Table 1), likely reducing the number of phosphorylation targets (Figure 4) and positively contributing to PaWRI2 protein stability.

These *in silico* analyses suggested many intriguing features that differentiate *Pa*WRI2 from *At*WRI2. Therefore, to confirm functionality *in vivo*, we performed transient expression assays in *N. benthamiana* leaves.

### Unlike *AtWRI2*, the Expression of *PaWRI2* Is Associated With Lipid Accumulation

Phylogenetic analyses of *WRI1* homologs indicate that *WRI2* has independently diverged from the remaining three orthologs, while *WRI3* and *WRI4* arose from a gene duplication event with *WRI1* and evolved independently (Supplementary Figure 1; Kilaru et al., 2015). Among the four *WRI1* orthologs in *Arabidopsis*, both *AtWRI3* and *AtWRI4*, but not *AtWRI2*, shared functional similarity with *AtWRI1* and were able to complement the *wri1* mutant and restore seed oil biosynthesis to near-wild type levels (To et al., 2012). However, the uniquely high expression levels of *PaWRI2* in avocado mesocarp during oil accumulation and distinct evolutionary separation from the remaining orthologs was intriguing (Kilaru et al., 2015). As such, to compare the functional role of *PaWRI2* to that of *PaWRI1* in oil accumulation, both genes were transiently expressed in *N. benthamiana* leaves. After seven days of co-infiltration of *Agrobacterium* expressing *PaWRI1* or *PaWRI2* and *P19*, a silencing−suppressor protein that enhances transgene expression (Naim et al., 2016), the leaves were harvested and analyzed for lipid accumulation. Interestingly, transient expression of both *PaWRI1* and *PaWRI2* resulted in a 7-fold increase in the number of LDs in *N. benthamiana* leaves (Figures 5A,B).

We further examined if these transcription factors affected fatty acid biosynthesis and thus lipid content in the leaf. Co-expression of *PaWRI2*/*P19* generated a 1.5-fold increase in the total lipid content, relative to leaves expressing *P19* alone (Figure 5C). The *PaWRI2*-mediated increase in lipid content, which was about 44% was greater than the 17-41% oil enhancement shown by the three transiently expressed isoforms of camelina *CsWRI1* in *N. benthamiana* leaves (An et al., 2017). Typically, *N. benthamiana* leaves contain about 0.2 to 0.3% lipids by FW of which only 20-30% is TAG. Ectopic expression studies using WRI1 orthologs have reported increase in total lipids of which 80-90% was TAG (An et al., 2017). *PaWRI1* expression resulted in higher LD numbers but only a smaller and statistically insignificant increase in total lipid content (Figure 5C).

Analysis of leaf total lipid fatty acid profile from samples expressing *PaWRI1* or *PaWRI2* further revealed a significant increase in 16:0 and decrease in 18:3 content, relative to the wild type leaves (Figure 5D). Although the role of WRI1 in oil accumulation has been unequivocally established, its ability to regulate fatty acid composition is not clear and is presumed to depend on the species, tissue type and its origin. For example, when camelina *CsWRI1* was expressed in *N. benthamiana* leaves, the content of both 18:1 and 18:2 increased and 18:3 decreased, while in transgenic seeds of stably transformed *Arabidopsis wri1* mutant plants, 18:3 content was highest and 18:1 remained low (An et al., 2017). Also, transient expression of *WRI1* homologs from potato, poplar, nutsedge, oat, and *Arabidopsis* showed reduced 18:0 and higher proportions of 18:1, relative to the control leaves (Grimberg et al., 2015). Interestingly, although avocado mesocarp lipids are highly enriched in monounsaturated fatty acid (oleic acid, 18:1) (Kikuta and Erickson, 1968; Kilaru et al., 2015), transient expression of neither *PaWRI* nor *PaWRI2* affected the oleic acid content in *N. benthamiana* leaves. Nevertheless, leaves expressing either *PaWRI* or *PaWRI2* showed a ∼1.4 − 1.7-fold increase in 16:0 and a ∼0.8 − 0.9-fold decrease in 18:3 levels. With ∼20% reduction in 18:3, this effect was higher with *PaWRI1* expression than with *PaWRI2*, where the decrease accounted for ∼10% (Figure 5D). Previous studies showed that transient expression of *WRI1* homologs in *N. benthamiana* leaves led to downregulation of the transcripts encoding plastidial fatty acid desaturase 7 (FAD7) that is responsible for the desaturation of 18:2 to 18:3, and long chain acyl-CoA synthetase 1 and 2 (LACS 1, 2), which catalyze the synthesis of long chain acyl-CoA that serves as a substrate for TAG assembly (Lu et al., 2009; Zhang et al., 2012; Grimberg et al., 2015). Whether *PaWRI1* and *PaWRI2* transient expression caused increased 16:0 and decreased 18:3 content due to changes in *FAD7*, *LACS1* and/or *LACS2* expression remains to be examined. Together, the transient expression studies implicate that both *PaWRI1* and *PaWRI2* are functional and able to induce lipid accumulation in non-seed tissues and also affect fatty acid composition (Figures 5B,C). *PaWRI2* enhanced lipid content and altered fatty acid composition more effectively than *PaWRI1*. These properties of *Pa*WRI2 are functionally unique and contrast sharply to that of *At*WRI2, which lacked any significant role in fatty acid biosynthesis (To et al., 2012). Considering the evolutionary position of avocado as a basal angiosperm, it is possible that *Pa*WRI2 is structurally unique and evolved as an early regulator of fatty acid biosynthesis, while such ability was lost in *At*WRI2 but retained by its paralogs.

## Conclusion

Avocado mesocarp is one of the most oleate-rich sources of oil in the plant kingdom. Detailed molecular knowledge of the regulatory machinery responsible for such sustained fatty acid biosynthetic and TAG assembly activity during the long fruit development period remains unresolved for non-seed tissues. Additionally, while the role for WRI1 as a master regulator of oil biosynthesis in oil-rich seed tissues in several species is well established, its significance in non-seed tissues remains unclear. Although the three WRI2 orthologs analyzed in this study contained only one AP2 domain, reflecting an ancestral feature, an interruption in the domain by a 35 amino acid stretch in *At*WRI2 was likely sufficient for loss of its function (Figure 1). Additionally, mutation in three amino acid residues in the AP2 domain of *At*WRI2 that are important for DNA binding might have also affected its function (Figure 1). In contrast, structurally unique characteristics of *Pa*WRI2, compared to its orthologs and paralogs, clarify its efficiency and functional novelty, despite containing only a single AP2 domain. *Pa*WRI2 lacked IDR3 (Figure 4 and Table 1) and the *C*-terminal PEST motif (Figure 4), which is expected to increase protein stability and sustained activity. Additionally, while disorder-promoting amino acid composition bias resulting in random coil secondary structure was inherent in all the orthologs (Supplementary Figure 4), *Pa*WRI2 displayed the highest disorder (Figure 2), suggesting maximum flexibility in interacting with target genes. Additionally, some conserved structural features such as the “VYL” micro exon and the KIN10 target residues (T70 and S166 identified in *At*WRI1) suggest that *Pa*WRI2 is involved in the maintenance of sugar-dependent lipid homeostasis in avocado mesocarp. Also, distinct phosphorylation targets that were predicted in the *C*-terminal region (Figure 4) might play a role in post-translational modifications, which can affect protein half-life, subcellular function, protein-protein interaction and homeostasis. Based on the previous RNA-seq analyses, it was clear that *Pa*WRI1, 2 and 3 are highly expressed during the extended period of oil accumulation. In our study, we show that *Pa*WRI2, unlike its ortholog *At*WRI2, was functional and induced oil accumulation in and altered the fatty acid profile of transiently infected leaf tissue more effectively than *Pa*WRI1 (Figure 5). Although the WRI2 orthologs formed a monophyletic clade as a result of independent divergence from their other paralogs, loss of function events among some orthologs were likely random. Bioinformatic analyses support the idea that all three mesocarp-expressed WRI paralogs, including *Pa*WRI2, are functional and likely contribute to coordinated and sustained oil biosynthesis. Further studies are pertinent to establish more details of the underlying regulatory network in oil biosynthesis in non-seed tissues in plants.

## Data Availability Statement

The original contributions presented in the study are available publicly at NCBI (https://www.ncbi.nlm.nih.gov/): *PaWRI* (MZ322905), *PaWRI2* (MZ322906), *PaWRI3* (MZ322907). The protein sequences used for data analyses are available publicly at NCBI (https://www.ncbi.nlm.nih.gov/): *At*WRI1 (NP_001030857.1), *At*WRI2 (NP_001189729.1), *At*WRI3 (NP_563990.1), *At*WRI4 (NP_178088.2), *Zm*WRI1a (NP_001137064.1), *Zm*WRI2 (NP_001145827.1), *Zm*WRI3/4b (XP_008651355.1), *Atr*WRI1 (XP_011620741.1), *Atr*WRI2 (XP_020526667.1), *Atr*WRI3 (XP_006845421.1), *Vv*WRI1 (XP_010659009.1), *Vv*WRI2 (XP_002284093.1), *Vv*WRI3 (XP_010652396.1), *Rc*WRI1 (NP_001310691.1), *Rc*WRI2 (XP_015581065.1), *Rc*WRI3 (NP_001310645.1), *Rc*WRI4 (XP_015573687.1), *Os*WRI1 (BAD68218.1), *Os*WRI2 (XP_015638088.1), *Os*WRI3 (XP_015617951.1), *Os*WRI4 (XP_015619972.1), *Br*WRI1 (XP_009116120.1), *Br*WRI2 (XP_009133508.1), *Br*WRI3 (XP_009149048.1), *Br*WRI4 (XP_009106692.1), *Eg*WRI1 (AHX71676.1), *Eg*WRI2 (XP_010938537.1), *Eg*WRI3 (XP_010912920.2), *Eg*WRI4 (XP_010914036.1), *Pt*WRI1 (XP_024465221.1), *Pt*WRI2 (XP_024458881.1), *Pt*WRI3 (XP_002297679.2), and *Pt*WRI4 (XP_024467077.1).

## Author Contributions

JB and SB conducted the bioinformatic analyses. SB and MR performed the cloning. MR conducted the transient expression assays. JB and MR performed the statistical analysis. JB, JS, and AK wrote the manuscript with assistance from MR and SB. All authors read and approved the final manuscript and contributed to the research design and data analyses.

## Disclaimer

Mention of trade names or commercial products in this publication is solely for the purpose of providing specific information and does not imply recommendation or endorsement from the United States Department of Agriculture. The USDA is an equal opportunity provider and employer.

## Conflict of Interest

The authors declare that the research was conducted in the absence of any commercial or financial relationships that could be construed as a potential conflict of interest.

## Supplementary Material

Supplementary Figure 1 Phylogenetic analysis and percentage identity among WRI1 homologs**. (A)** Phylogenetic analysis of WRI1 homologs in *Arabidopsis thaliana (At)*, *Zea mays (Zm)*, *Amborella trichopoda (Atr)*, *Populus trichocarpa (Pt)*, *Ricinus communis (Rc)*, *Brassica rapa (Br)*, *Elaeis guineensis (Eg)*, *Vitis vinifera (Vv)*, *Oryza sativa (Os)* and *Persea americana (Pa)* was carried out using UPGMA method (see Supplementary Table 1). An AP2-domain transcription factor from *Chlamydomonas reinhardtii* (*Cr*AP2) was used as outgroup. Percentages indicate bootstrap values for 1000 replicates. The WRI-orthologs are shown in color; WRI1 (black), WRI2 (pink), WRI3 (orange), WRI4 (blue). **(B)** Percentage of amino acid sequence identity among the WRI1 homologs from avocado (*Pa*), Arabidopsis (*At*), and maize (*Zm*).

Supplementary Figure 2 Sequence alignment of WRI1, WRI2, and WRI3 from various species showing conserved amino acids. Multiple sequence alignment of primary sequences of WRI1, 2, and 3 from species as described in Supplementary Figure 1 are shown in a color-coded format; red and blue colors represent the most (10, indicated by asterisk) and least (0) conserved regions, respectively on a scale of 0 to 10. Positions of the AP2 domains are shown as pink color bars under the sequences and the amino acid residues in the AP2 domain important for DNA interaction are represented by (▲).

Supplementary Figure 3 Multiple sequence alignment of WRI1 homologs from the select three species. The alignment shows KIN10 target sites (red box) in the WRI1 homologs with reference to T70 and S166 identified in *At*WRI1. The S166 residue is mutated in *At*WRI2 and *Zm*WRI2. The highlighted amino acid residues correspond to the PEST motifs.

Supplementary Figure 4 Amino acid composition profile of WRI1 homologs. Amino acid composition profile of WRI1 homologs showing the enriched and depleted amino acids as compared to SwissProt51 database. The amino acids are arranged in the increasing order of hydrophobic character on the *x*-axis and the *y*-axis represents the composition profile. The values >0 represents an enrichment and <0 represents a depletion of corresponding amino acids in the protein sequence. The significantly (*p* < 0.05) enriched (green) and depleted (blue) residues are denoted in color.

Supplementary Figure 5 Graphical representation of the predicted IDRs. The predicted IDRs in all the WRI homologs are graphically plotted with the amino acid position on the *x*-axis and the PONDR score on the *y*-axis. Regions showing PONDR score > 0.5 are considered as IDRs. For *Atr*WRI1 and *Atr*WRI2, although the figures are not showing the PONDR score, the predicted IDRs by the same software are as follows: 30-53, 137-160, 234-348 for WRI1 and 12-84, 168-190 for WRI2. Both of them have the *C*-terminal IDR.

Supplementary Table 1 List of online tools used for various predictive structural analysis of WRI1 orthologs.
