# Supplementary material for: Functional and Predictive Structural Characterization of WRINKLED2, A Unique Oil Biosynthesis Regulator in Avocado
Source: Front Plant Sci. 2021 Jun 8;12:648494. doi: 10.3389/fpls.2021.648494 (PMC8218904; doi:10.3389/fpls.2021.648494)
Supplement: Supplementary Figure 1 — Phylogenetic analysis and percentage identity among WRI1 homologs. (A) Phylogenetic analysis of WRI1 homologs in Arabidopsis thaliana (At), Zea mays (Zm), Amborella trichopoda (Atr), Populus trichocarpa (Pt), Ricinus communis (Rc), Brassica rapa (Br), Elaeis guineensis (Eg), Vitis vinifera (Vv), Oryza sativa (Os) and Persea americana (Pa) was carried out using UPGMA method (see Supplementary Table 1). An AP2-domain transcription factor from Chlamydomonas reinhardtii (CrAP2) was used as outgroup. Percentages indicate bootstrap values for 1000 replicates. The WRI-orthologs are shown in color; WRI1 (black), WRI2 (pink), WRI3 (orange), WRI4 (blue). (B) Percentage of amino acid sequence identity among the WRI1 homologs from avocado (Pa), Arabidopsis (At), and maize (Zm). [file Data_Sheet_1.PDF]

Supplementary material

Supplementary Table 1. List of online tools used for various predictive structural analysis of WRI1 orthologs

Analysis	Tool
Protein sequence prediction	ExPASy Translate Tool
Multiple sequence alignment	PRALINE
Amino acid profile	COMPOSITION PROFILER
Intrinsic disordered region prediction	PONDR-VL3
Secondary structure prediction	NPS@SOPMA
Phosphorylation site prediction	NetPhos 3.1
PEST motif prediction	epestfind

Supplementary Figure 1.

(A)

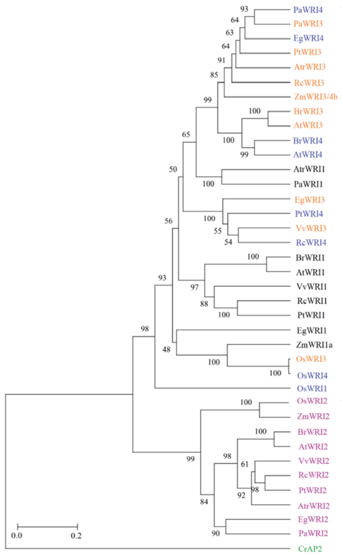

(B)

Protein	*Pa*WRI1	*At*WRI1	*Zm*WRI1a	*Pa*WRI2	*At*WRI2	*Zm*WRI2	*Pa*WRI3	*At*WRI3	*Zm*WRI3/4b	*Pa*WRI4	*At*WRI4
***Pa*WRI1**	100										
***At*WRI1**	56.7	100									
***Zm*WRI1a**	54.5	50.4	100								
***Pa*WRI2**	39.2	39.2	41.3	100							
***At*WRI2**	35.4	36.3	39.0	57.2	100						
***Zm*WRI2**	38.3	38.5	39.0	55.5	51.7	100					
***Pa*WRI3**	57.8	53.3	51.0	41.5	38.6	40.3	100				
***At*WRI3**	54.4	50.7	52.2	37.3	35.0	35.3	66.1	100			
***Zm*WRI3/4b**	49.6	49.2	49.8	40.1	34.8	37.6	69.7	58.7	100		
***Pa*WRI4**	62.4	57.2	57.1	44.5	40.5	41.6	77.6	64.4	68.6	100	
***At*WRI4**	54.6	54.1	52.0	39.8	39.8	38.7	65.9	70.5	55.5	64.8	100

Supplementary Figure 1. Phylogenetic analysis and percentage identity among WRI1 homologs

A) Phylogenetic analysis of WRI1 homologs in *Arabidopsis thaliana (At)*, *Zea mays (Zm)*, *Amborella trichopoda (Atr)*, *Populus trichocarpa (Pt)*, *Ricinus communis (Rc)*, *Brassica rapa (Br)*, *Elaeis guineensis (Eg)*, *Vitis vinifera (Vv)*, *Oryza sativa (Os)* and *Persea americana (Pa)* was carried out using UPGMA method (see Table S1). An AP2-domain transcription factor from *Chlamydomonas reinhardtii* (*Cr*AP2) was used as outgroup. Percentages indicate bootstrap values for 1000 replicates. The WRI-orthologs are shown in color; WRI1 (black), WRI2 (pink), WRI3 (orange), WRI4 (blue). **B)** Percentage of amino acid sequence identity among the WRI1 homologs from avocado (*Pa*), Arabidopsis (*At*), and maize (*Zm*).

Supplementary Figure 2.

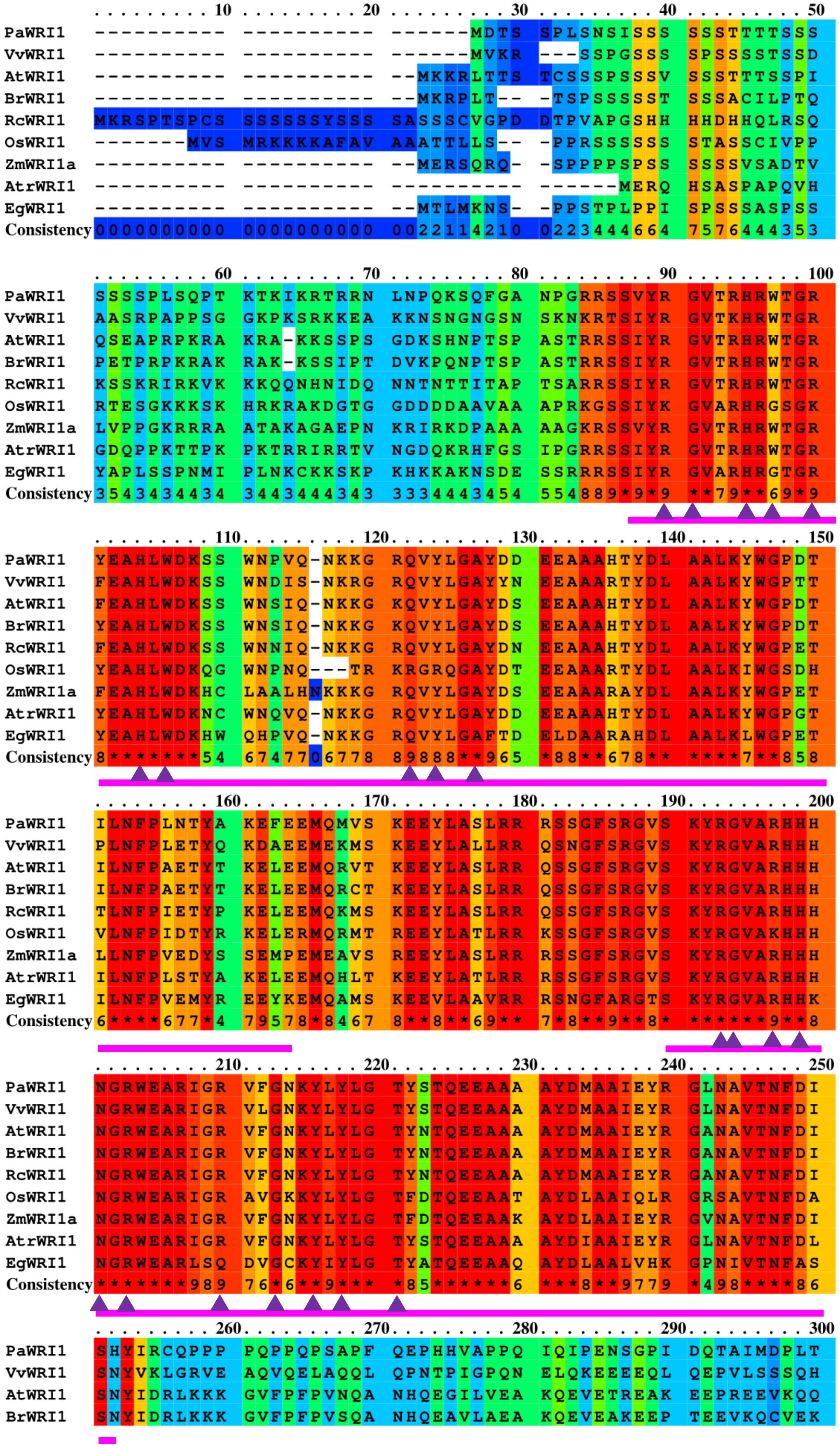

```
RcWRI1      SNYIDRLKKK GILLDQILPD QPLRKCSSES EEAEAEAEVE RLPSLPSSIL
OsWRI1      SCYTYTDHLP PPPPPQPPSV CKTEPELEPP QPAAPPGSES LLRPKMEPCD
ZmWRI1a     SCYLDHPLFL AQLQQEPQVV PALNQEPQPD QSETGTTEQE PESSEAKTPD
AtrWRI1     SHYIRSPFPQ PTYTFEDSQT PKFPMMASTN FQIPNSDLCD AMVGPEFPSE
EgWRI1      SVYKHRLQPF MQLLVKPETE PAQEDLGVMQ MEATETIDQT MPNYDLPEIS
Consistency *3*6433333 4434343443 3334343444 4543543543 3433332433

            .........310.........320.........330.........340.........350
PaWRI1      DQPWNLCMDP TFNSIPVP-- ---------- ---------- ----------
VvWRI1      LPSMDSSAME IMDPADDP-- ---------- ---------- ----------
AtWRI1      YVEEPPQEEE EKEEEKAEQQ EAEIVGYSEE AAVVNCCIDS STIMEMDRCG
BrWRI1      EEPQEAKEEK TEKKQQQQEV EEAVV----- ----TCCIDS SESN------
RcWRI1      PQEQDTISPQ LQCTQLLPSM DSCTM----- -----INMDP IEDN------
OsWRI1      DWEPPAICPS LRDADDADHA IAEIL----- ----PALCMD RA--------
ZmWRI1a     GSAEPDENAV PDDTAEPLTT VDDSIE---- ---------- ----------
AtrWRI1     QTWAPQVDCS FNGLP----- ---------- ---------- ----------
EgWRI1      WTFDIDHDLG AYPLLDVP-- ---------- ---------- ----------
Consistency 2333433323 3333332311 1111200000 0000000000 0000000000

            .........360.........370.........380.........390.........400
PaWRI1      ---NIALDKS IELHDLF--N G-----TGFE DNIEFLFDGA EGGFPTGPTT
VvWRI1      ---DLPWNFC AYSTLLVPDV P-----LGKG GELSDLFYEK GFEDNIDYMF
AtWRI1      DNNELAWNFC MMDTGFSPFL TDQNLANENP IEYPELFNEL AFEDNIDFMF
BrWRI1      ---ELAWDFC MMDSGFAPFL TDSNLSSENP IEYPELFNEM GFEDNIDFMF
RcWRI1      ---ELTWSFC L-DSGLTLFS VPE-LPLENA CELPDLFDDT GFEDNIDLIF
OsWRI1      ---DFEARYP ARRARDAAAD GWS--TSSDD VAAASVDDDV LRSLPDDVGF
ZmWRI1a     ---EGLWSPC MDYELDTMSR P----NFGSS INLSEWFADA DFDCNIGCLF
AtrWRI1     ---------- ---------- ---------- --DSTTFED- -----IDFLF
EgWRI1      ---------- ---------- --------IE DDQHDILNDL NFEGNIEHLF
Consistency 0004323323 3112122111 2000012223 2434566463 2442566358

            .........410.........420.........430.........440.........450
PaWRI1      TGIHECEGNE MKLDDGIFEN SGGFEGDFLN DIEEGGECGL IVEN-----M
VvWRI1      EGAAGNEEE- ------SNSA ENGVKENGLS SSSSSSSGCD SASP-----A
AtWRI1      DDGKHECLNL ENLD-CC--- -VVGR-ESPP -SSSSPLSCL STD-------
BrWRI1      EEGKQDCLSL ENLD-CCDGV VVVGR-ESPT SLSSSPLSCL STD-------
RcWRI1      DGCCFGN-DD DGGG-GANHQ EFMVESRGCR VGEVGISGSM EEENGKEMCC
OsWRI1      VDDVESLFLD APGP-AAAAA AAAMPDDVER AVQRAPSAAS RRA-------
ZmWRI1a     DGCSAAD--- -------EGS KDGVGLADFS LFEA--GDVQ LKDV-----L
AtrWRI1     DNSQEEGVSE -----EKEAK GNVDNTLQAS GSSEGGREAM GLNHG----D
EgWRI1      EEFETFG--- ---------- GNESGSDGFS ASKGA----- ----------
Consistency 5523332121 1111012222 3333324325 3464423333 2340000000

            .........460.........470
PaWRI1      NASVDSISYP LPISICS---
VvWRI1      STT-TLASCT YSM-------
AtWRI1      SASSTT-TTT TSVS-CNYLV
BrWRI1      SASSTTTTTI TSVS-CNYSV
RcWRI1      SSSSPSCSTT TSVSCCNYSV
OsWRI1      NAAAVS-YAI SSLASGRWWY
ZmWRI1a     SDMEEGIQPP AMISVCN---
AtrWRI1     MSSMNSISYP CPISICS---
EgWRI1      ---------- ----------
Consistency 5452342423 3465143101
```

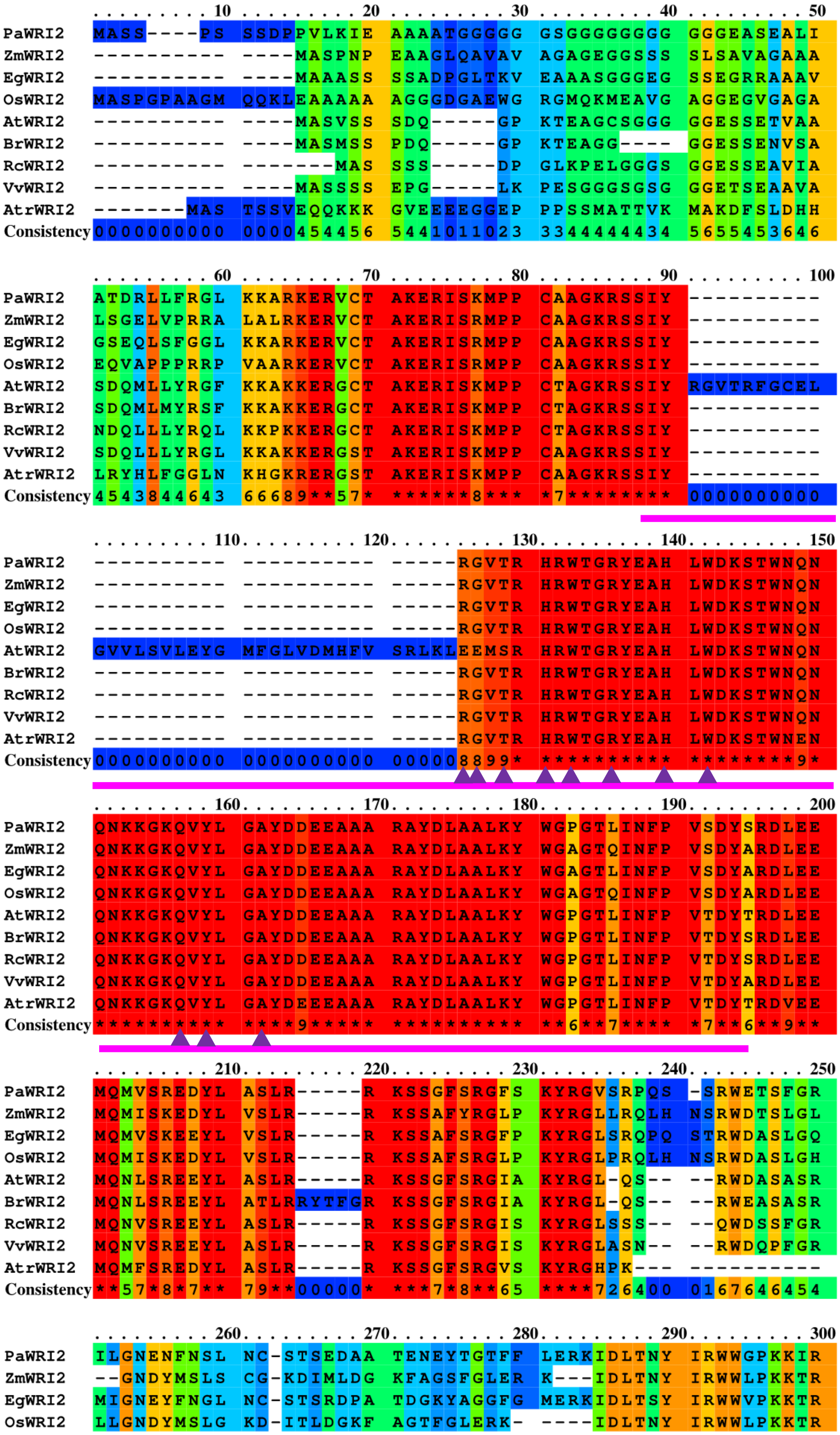

. . . . 10 . . . . 20 . . . . 30 . . . . 40 50
PaWRI2 MASS----PS SSDPPVLKIE AAAATGGGGG GSGGGGGGGG GGGEASEALI
ZmWRI2 ---------- ----MASPNP EAAGLQAVAV AGAGEGGSSS SLSAVAGAAA
EgWRI2 ---------- ----MAAASS SSADPGLTKV EAAASGGGEG SSEGRRAAAV
OsWRI2 MASPGPAAGM QQKLEAAAAA AGGGDGAEWG RGMQKMEAVG AGGEGVGAGA
AtWRI2 ---------- ----MASVSS SDQ-----GP KTEAGCSGGG GGESSETVAA
BrWRI2 ---------- ----MASMSS PDQ-----GP KTEAGG---- GGESSENVSA
RcWRI2 ---------- -------MAS SSS-----DP GLKPELGGGS GGESSEAVIA
VvWRI2 ---------- ----MASSSS EPG-----LK PESGGGSGSG GGETSEAAVA
AtrWRI2 -------MAS TSSVEQQKKK GVEEEEGGEP PPSSMATTVK MAKDFSLDHH
Consistency 0000000000 0000454456 5441011023 3344444434 5655453646
. . . . 60 . . . . 70 . . . . 80 . . . . 90 100
PaWRI2 ATDRLLFRGL KKARKERVCT AKERISKMPP CAAGKRSSIY ----------
ZmWRI2 LSGELVPRRA LALRKERVCT AKERISRMPP CAAGKRSSIY ----------
EgWRI2 GSEQLSFGGL KKARKERVCT AKERISRMPP CAAGKRSSIY ----------
OsWRI2 EQVAPPPRRP VAARKERVCT AKERISRMPP CAAGKRSSIY ----------
AtWRI2 SDQMLLYRGF KKAKKERGCT AKERISKMPP CTAGKRSSIY RGVTRFGCEL
BrWRI2 SDQMLMYRSF KKAKKERGCT AKERISKMPP CTAGKRSSIY ----------
RcWRI2 NDQLLLYRQL KKPKKERGCT AKERISKMPP CTAGKRSSIY ----------
VvWRI2 SDQLLLYRGL KKAKKERGST AKERISKMPP CAAGKRSSIY ----------
AtrWRI2 LRYHLFGGLN KHGKRERGST AKERISKMPP CAAGKRSSIY ----------
Consistency 4543844643 66689**57* ******8*** *7******** 0000000000
. . . . 110 . . . . 120 . . . . 130 . . . . 140 150
PaWRI2 ---------- ---------- -----RGVTR HRWTGRYEAH LWDKSTWNQN
ZmWRI2 ---------- ---------- -----RGVTR HRWTGRYEAH LWDKSTWNQN
EgWRI2 ---------- ---------- -----RGVTR HRWTGRYEAH LWDKSTWNQN
OsWRI2 ---------- ---------- -----RGVTR HRWTGRYEAH LWDKSTWNQN
AtWRI2 GVVLSVLEYG MFGLVDMHFV SRLKLEEMSR HRWTGRYEAH LWDKSTWNQN
BrWRI2 ---------- ---------- -----RGVTR HRWTGRYEAH LWDKSTWNQN
RcWRI2 ---------- ---------- -----RGVTR HRWTGRYEAH LWDKSTWNQN
VvWRI2 ---------- ---------- -----RGVTR HRWTGRYEAH LWDKSTWNQN
AtrWRI2 ---------- ---------- -----RGVTR HRWTGRYEAH LWDKSTWNEN
Consistency 0000000000 0000000000 000008899* ********** ********9*
. . . . 160 . . . . 170 . . . . 180 . . . . 190 200
PaWRI2 QNKKGKQVYL GAYDDEEAAA RAYDLAALKY WGPGTLINFP VSDYSRDLEE
ZmWRI2 QNKKGKQVYL GAYDDEEAAA RAYDLAALKY WGAGTQINFP VSDYARDLEE
EgWRI2 QNKKGKQVYL GAYDDEEAAA RAYDLAALKY WGAGTLINFP VSDYARDLEE
OsWRI2 QNKKGKQVYL GAYDDEEAAA RAYDLAALKY WGAGTQINFP VSDYARDLEE
AtWRI2 QNKKGKQVYL GAYDDEEAAA RAYDLAALKY WGPGTLINFP VTDYTRDLEE
BrWRI2 QNKKGKQVYL GAYDDEEAAA RAYDLAALKY WGPGTLINFP VTDYSRDLEE
RcWRI2 QNKKGKQVYL GAYDDEEAAA RAYDLAALKY WGPGTLINFP VTDYSRDLEE
VvWRI2 QNKKGKQVYL GAYDDEEAAA RAYDLAALKY WGPGTLINFP VTDYARDLEE
AtrWRI2 QNKKGKQVYL GAYDEEEAAA RAYDLAALKY WGPGTLINFP VTDYTRDVEE
Consistency ********** ****9***** ********** **6**7**** *7**6**9**
. . . . 210 . . . . 220 . . . . 230 . . . . 240 250
PaWRI2 MQMVSREDYL ASLR-----R KSSGFSRGFS KYRGVSRPQS -SRWETSFGR
ZmWRI2 MQMISKEDYL VSLR-----R KSSAFYRGLP KYRGLLRQLH NSRWDTSLGL
EgWRI2 MQMVSKEEYL VSLR-----R KSSAFSRGFP KYRGLSRQPQ STRWDASLGQ
OsWRI2 MQMISKEDYL VSLR-----R KSSAFSRGLP KYRGLPRQLH NSRWDASLGH
AtWRI2 MQNLSREEYL ASLR-----R KSSGFSRGIA KYRGL-QS-- --RWDASASR
BrWRI2 MQNLSREEYL ATLRRYTFGR KSSGFSRGIA KYRGL-QS-- --RWEASASR
RcWRI2 MQNVSREEYL ASLR-----R KSSGFSRGIS KYRGLSSS-- --QWDSSFGR
VvWRI2 MQNVSREEYL ASLR-----R KSSGFSRGIS KYRGLASN-- --RWDQPFGR
AtrWRI2 MQMFSREDYL ASLR-----R KSSGFSRGVS KYRGHPK--- ----------
Consistency **57*8*7** 79**00000* ***7*8**65 ****726400 0167646454
. . . . 260 . . . . 270 . . . . 280 . . . . 290 300
PaWRI2 ILGNENFNSL NC-STSEDAA TENEYTGTFF LERKIDLTNY IRWWGPKKIR
ZmWRI2 --GNDYMSLS CG-KDIMLDG KFAGSFGLER K---IDLTNY IRWWLPKKTR
EgWRI2 MIGNEYFNGL NC-STSRDPA TDGKYAGGFG MERKIDLTSY IRWWVPKKTR
OsWRI2 LLGNDYMSLG KD-ITLDGKF AGTFGLERK- ----IDLTNY IRWWLPKKTR

```
AtWRI2      MPGPEYFSNI HY-GAGDDRG TEGDFLGSFC LERKIDLTGY IKWWGANKNR
BrWRI2      MPGPEYFSNL HY-GAGDERG AEGDFLGSFC LERKIDLTGY IKWWGVNKSR
RcWRI2      MPGSEYFSSI NYGAADDPAA ESEYVGSLCF ERKI-DLTSY IRWWGFNKTR
VvWRI2      IAGQEYFNNM HY-GMGDDAA AESELFGGFC MERKIDLTGY IKWWGPNKTR
AtrWRI2     ---------- ---------- ---------- ---------- ----------
Consistency 4273665533 3203324334 4332235231 3332577747 7677335747

            .........310.........320.........330.........340.........350
PaWRI2      RSDPITKSSD ETHGISDAGS ELKTFEWPSQ HTEPYQLPSL GLSCKGKP--
ZmWRI2      QSDTSKTEEI ADE------- -IRAIESSMQ QTEPYKLPSL GFSSPSKP--
EgWRI2      QSESTSNADV GRE------- -LKTLECSIQ PTEPYQLPSL GLPREGKP--
OsWRI2      QSDTSKMEEV TDE------- -IRAIESSMQ RTEPYKFPSL GLHSNSKP--
AtWRI2      QPESSSKASE DAN-VEDAGT ELKTLEHTSH ATEPYKAPNL GVLCGTQRKE
BrWRI2      QPESSSKASE DAK-VEDAGT ELKALEHTSQ ATEPYKAPNL GVLQRKGKQI
RcWRI2      ESVSKSSDER KHG-YGEDIS ELKSSEWAVQ STEPYQMPRL GMPDNGKKH-
VvWRI2      QSDSLAKSSE ETK-QGSGED IGSELKTLEW AIQPTEPYQM PRLGLPHEGK
AtrWRI2     ---------- ---------- ---------- ---------- ----------
Consistency 6545344453 3230111111 1554462335 3677653647 6422234300

            .........360.........370.........380.........390.........400
PaWRI2      ----FASAMS ACRILAQSAA FKKMQEKASE AQDGEHTN-N IGHE------
ZmWRI2      ----SSMGLS ACSILSQSDA FKSFLEKSTK LSEECSLS-K EIVE------
EgWRI2      ----RAQGLS ACSILSKSEA FKNFVEKSSK TSEMKDDTSN KEMD------
OsWRI2      ----SSVVLS ACDILSQSDA FKSFSEKSTK LSEECTFS-K EMDE------
AtWRI2      KEISSPSSSS ALSILSQSPA FKSLEEK--- VLKI---QES CNNENDENAN
BrWRI2      T--SPSSTSS ALSILSASPA YKSMEEKVMK IQESSSTREN DENANRNITS
RcWRI2      ----KCSKIS ALSILSHSAA YKNLQEKASK KQENCTDNDE KEN---KKTN
VvWRI2      K--QKSSTVS ALSILSRSAA YKSLEEKASK KQENNTENDE NEN--KNKIN
AtrWRI2     ---------- ---------- ---------- ---------- ----------
Consistency 0000344347 7457774737 6754377335 3462231414 2332000001

            .........410.........420.........430.........440.........450
PaWRI2      KTVLKLS--S VGGSESSGVG LSLG------ -EMPLQKTSY PLGPFLSAPL
ZmWRI2      GKTVASV--P ATGYDTGAIN INMN------ -ELLVQRSTY SMAPVMPTPM
EgWRI2      HGKADPLLFS GGGLDRSGVT LGLS------ -ELVVQRTPY SFSPLLSAPL
OsWRI2      GKTVTPV--P ATGHDTTAVN MNVN------ -GLLVQRAPY TL-PSVTAQM
AtWRI2      RNIINMEKNN GKAIEKPVVS HGVALGG--A AALSLQKSMY PLTSLLTAPL
BrWRI2      IEKSHGKEIE KPAV-SHGVA LGSGGGVAPA AALSLQKSMY PLSSLLTAPL
RcWRI2      KMDYGKAVEK STSHDGSNER LGAALGM--S GGLSLQRNAY QLAPFLSAPL
VvWRI2      KMDHGKAVEK STSHDSGSER LGVALGL--G GEFSIQRSVY PLTPLLSAPL
AtrWRI2     ---------- ---------- ---------- ---------- ----------
Consistency 2223223103 3342433343 5544010000 1363676537 4635465666

            .........460.........470.........480.........490.........500
PaWRI2      LTNCSNIDPS PDSAFWTNL- -IQPTGLSLS TTHRKNEISS ------TYTF
ZmWRI2      KSTWSPADPS VDPLFWSNF- -VLPSSQPVT MATITTTTFA KNEVSSSDPF
EgWRI2      RTSWNSVDPV PDPVFWTSL- -VSPSEQSMT TTFRKNDVSS ------SYSY
OsWRI2      KNTWNPADPS ADPLFWTNF- -ILPASQPVT MATIATTTFA KNEVSSSDPF
AtWRI2      LTNYNTLDPL ADPILWT--- PFLPSGSSLT SEVTKTETSC -----STYSY
BrWRI2      LSNYNALDPL GDRILWT--- PFLPPGSSHT SEVTKTETSC -----STYSY
RcWRI2      LTNYNAIDPL VDPILWTSLV PVLPAGFSRN SEVTKTETS- -----STYTF
VvWRI2      LTNYSSVDPL ADPILWTSLV PALPTGLPRT AEVTKTETS- -----STYTF
AtrWRI2     ---------- ---------- ---------- ---------- ----------
Consistency 4554535774 3755576320 1457443436 4433555552 0000046446

            ......
PaWRI2      FRQE--
ZmWRI2      QSQE--
EgWRI2      QCPD--
OsWRI2      HGQE--
AtWRI2      LPQEK-
BrWRI2      LPQEK-
RcWRI2      FRPEE-
VvWRI2      FHQEEQ
AtrWRI2     ------
Consistency 325710
```

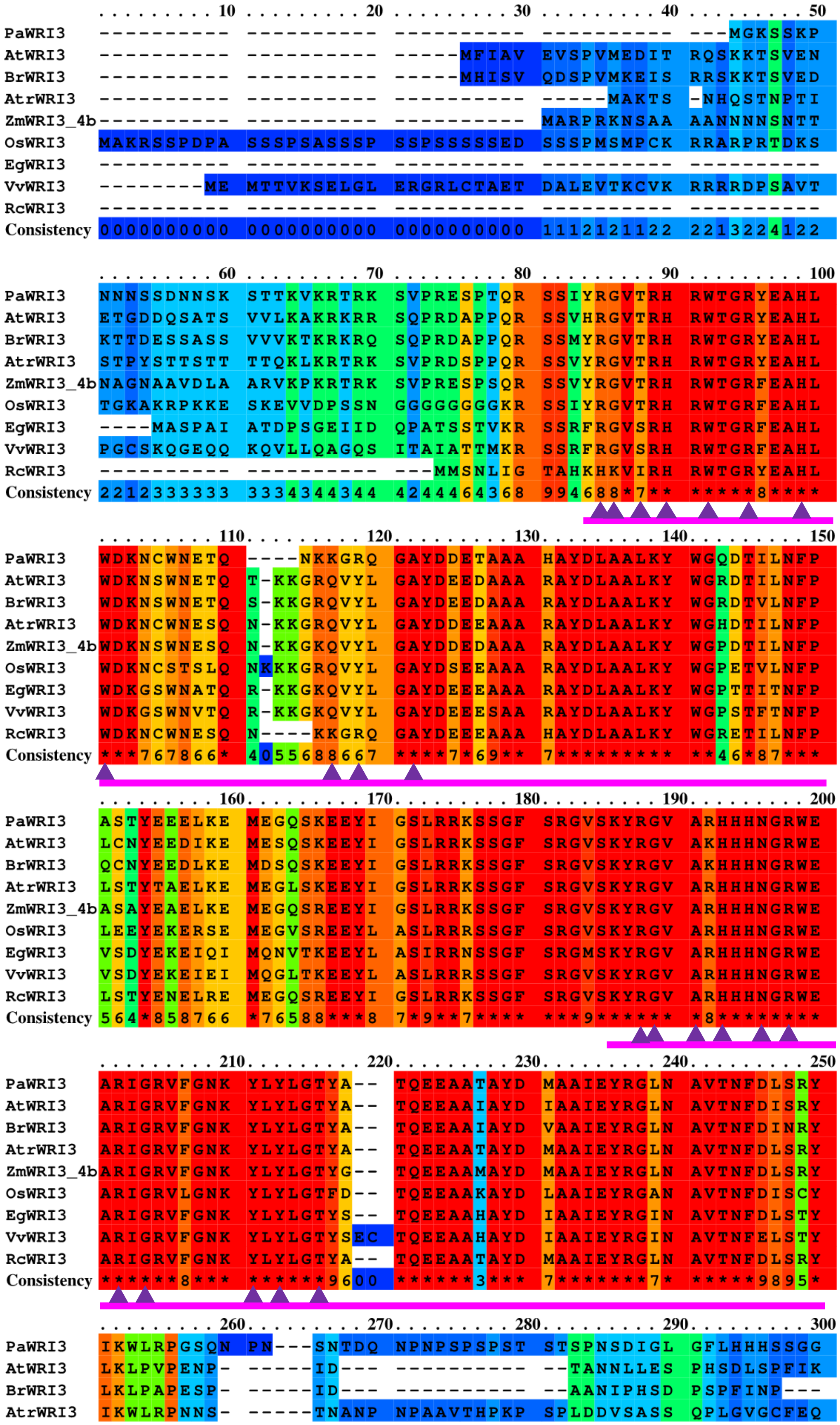
. . . . 10 . . . . 20 . . . . 30 . . . . 40 50
PaWRI3 ---------- ---------- ---------- ---------- ---MGKSSKP
AtWRI3 ---------- ---------- -----MFIAV EVSPVMEDIT RQSKKTSVEN
BrWRI3 ---------- ---------- -----MHISV QDSPVMKEIS RRSKKTSVED
AtrWRI3 ---------- ---------- ---------- -----MAKTS -NHQSTNPTI
ZmWRI3_4b ---------- ---------- ---------- MARPRKNSAA AANNNNSNTT
OsWRI3 MAKRSSPDPA SSSPSASSSP SSPSSSSSED SSSPMSMPCK RRARPRTDKS
EgWRI3 ---------- ---------- ---------- ---------- ----------
VvWRI3 --------ME MTTVKSELGL ERGRLCTAET DALEVTKCVK RRRRDPSAVT
RcWRI3 ---------- ---------- ---------- ---------- ----------
Consistency 0000000000 0000000000 0000000000 1112121122 2213224122
. . . . 60 . . . . 70 . . . . 80 . . . . 90 100
PaWRI3 NNNSSDNNSK STTKVKRTRK SVPRESPTQR SSIYRGVTRH RWTGRYEAHL
AtWRI3 ETGDDQSATS VVLKAKRKRR SQPRDAPPQR SSVHRGVTRH RWTGRYEAHL
BrWRI3 KTTDESSASS VVVKTKRKRQ SQPRDAPPQR SSMYRGVTRH RWTGRYEAHL
AtrWRI3 STPYSTTSTT TTQKLKRTRK SVPRDSPPQR SSVYRGVTRH RWTGRYEAHL
ZmWRI3_4b NAGNAAVDLA ARVKPKRTRK SVPRESPSQR SSVYRGVTRH RWTGRFEAHL
OsWRI3 TGKAKRPKKE SKEVVDPSSN GGGGGGGGKR SSIYRGVTRH RWTGRFEAHL
EgWRI3 ----MASPAI ATDPSGEIID QPATSSTVKR SSRFRGVSRH RWTGRFEAHL
VvWRI3 PGCSKQGEQQ KQVLLQAGQS ITAIATTMKR SSRFRGVSRH RWTGRFEAHL
RcWRI3 ---------- ---------- ---MMSNLIG TAHKHKVIRH RWTGRYEAHL
Consistency 2212333333 3334344344 4244464368 994688*7** *****8****
. . . . 110 . . . . 120 . . . . 130 . . . . 140 150
PaWRI3 WDKNCWNETQ ----NKKGRQ GAYDDETAAA HAYDLAALKY WGQDTILNFP
AtWRI3 WDKNSWNETQ T-KKGRQVYL GAYDEEDAAA RAYDLAALKY WGRDTILNFP
BrWRI3 WDKNSWNETQ S-KKGRQVYL GAYDEEDAAA RAYDLAALKY WGRDTVLNFP
AtrWRI3 WDKNCWNESQ N-KKGRQVYL GAYDDEEAAA RAYDLAALKY WGHDTILNFP
ZmWRI3_4b WDKNSWNESQ N-KKGKQVYL GAYDDEDAAA RAYDLAALKY WGPDTILNFP
OsWRI3 WDKNCSTSLQ NKKKGRQVYL GAYDSEEAAA RAYDLAALKY WGPETVLNFP
EgWRI3 WDKGSWNATQ R-KKGKQVYL GAYDEEEAAA RAYDLAALKY WGPTTITNFP
VvWRI3 WDKGSWNVTQ R-KKGKQVYL GAYDEEESAA RAYDLAALKY WGPSTFTNFP
RcWRI3 WDKNCWNESQ N----KKGRQ GAYDEEEAAA HAYDLAALKY WGRETILNFP
Consistency ***767866* 4055688667 ****7*69** 7********* **46*87***
. . . . 160 . . . . 170 . . . . 180 . . . . 190 200
PaWRI3 ASTYEEELKE MEGQSKEEYI GSLRRKSSGF SRGVSKYRGV ARHHHNGRWE
AtWRI3 LCNYEEDIKE MESQSKEEYI GSLRRKSSGF SRGVSKYRGV AKHHHNGRWE
BrWRI3 QCNYEEDLKE MDSQSKEEYI GSLRRKSSGF SRGVSKYRGV AKHHHNGRWE
AtrWRI3 LSTYTAELKE MEGLSKEEYI GSLRRKSSGF SRGVSKYRGV ARHHHNGRWE
ZmWRI3_4b ASAYEAELKE MEGQSREEYI GSLRRKSSGF SRGVSKYRGV ARHHHNGRWE
OsWRI3 LEEYEKERSE MEGVSREEYL ASLRRRSSGF SRGVSKYRGV ARHHHNGRWE
EgWRI3 VSDYEKEIQI MQNVTKEEYL ASIRRNSSGF SRGMSKYRGV ARHHHNGRWE
VvWRI3 VSDYEKEIEI MQGLTKEEYL ASLRRRSSGF SRGVSKYRGV ARHHHNGRWE
RcWRI3 LSTYENELRE MEGQSREEYI GSLRRKSSGF SRGVSKYRGV ARHHHNGRWE
Consistency 564*858766 *76588***8 7*9**7**** ***9****** *8********
. . . . 210 . . . . 220 . . . . 230 . . . . 240 250
PaWRI3 ARIGRVFGNK YLYLGTYA-- TQEEAATAYD MAAIEYRGLN AVTNFDLSRY
AtWRI3 ARIGRVFGNK YLYLGTYA-- TQEEAAIAYD IAAIEYRGLN AVTNFDISRY
BrWRI3 ARIGRVFGNK YLYLGTYA-- TQEEAAIAYD VAAIEYRGLN AVTNFDINRY
AtrWRI3 ARIGRVFGNK YLYLGTYA-- TQEEAATAYD MAAIEYRGLN AVTNFDLSRY
ZmWRI3_4b ARIGRVFGNK YLYLGTYG-- TQEEAAMAYD MAAIEYRGLN AVTNFDLSRY
OsWRI3 ARIGRVLGNK YLYLGTFD-- TQEEAAKAYD LAAIEYRGAN AVTNFDISCY
EgWRI3 ARIGRVFGNK YLYLGTYS-- TQEEAAHAYD IAAIEYRGIN AVTNFDLSTY
VvWRI3 ARIGRVFGNK YLYLGTYSEC TQEEAAHAYD IAAIEYRGIN AVTNFELSTY
RcWRI3 ARIGRVFGNK YLYLGTYA-- TQEEAATAYD MAAIEYRGLN AVTNFDLSRY
Consistency ******8*** ******9600 ******3*** 7*******7* *****9895*
. . . . 260 . . . . 270 . . . . 280 . . . . 290 300
PaWRI3 IKWLRPGSQN PN---SNTDQ NPNPSPSPST STSPNSDIGL GFLHHHSSGG
AtWRI3 LKLPVPENP- -----ID--- ---------- --TANNLLES PHSDLSPFIK
BrWRI3 LKLPAPESP- -----ID--- ---------- --AANIPHSD PSPFINP---
AtrWRI3 IKWLRPNNS- -----TNANP NPAAVTHPKP SPLDDVSASS QPLGVGCFEQ

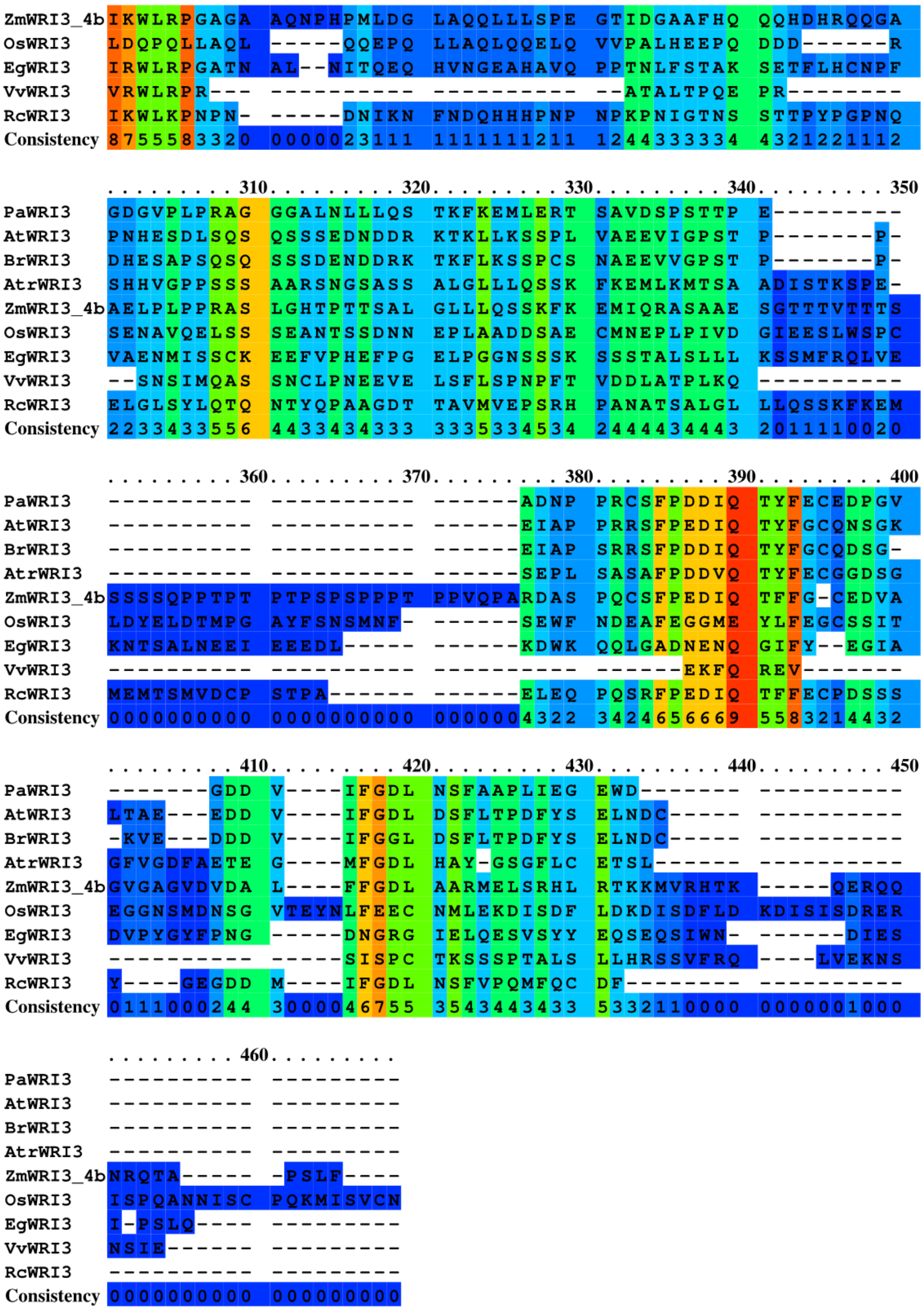

Supplementary Figure 2: Sequence alignment of WRI1, WRI2, and WRI3 from various species showing conserved amino acids

Multiple sequence alignment of primary sequences of WRI1, 2, and 3 from species as described in Supplementary Figure 1 are shown in a color-coded format; red and blue colors represent the most (10, indicated by asterisk) and least (0) conserved regions, respectively on a scale of 0 to 10. Positions of the AP2 domains are shown as pink color bars under the sequences and the amino acid residues in the AP2 domain important for DNA interaction are represented by (▲).

Supplementary Figure 3.

```
ZmWRI2          ----------MAS----PNPEAAGLQAVAVAGAG----------EGGSSSSLSAVAGAAA 36
PaWRI2          ----------MASSPSSSDPPVLKIEAAAATGGGGGGSGGGGGGGGGGGEASE---ALIA 47
AtWRI2          ----------MASV----------------SSSDQGPKTEAGCSGGGGGESSE---TVAA 31
ZmWRI1a         ----------MERSQRQSPP---------------------PPSPSSSSSSVS-ADTVLV 28
ZmWRI3/4b       ----------MARPRKNSAA---------------------AANNN-----NS-NTTNAG 23
PaWRI3          ----------------MGKS---------------------S----------K-PNNNSS 12
AtWRI3          MFIAVEVSPVMEDITRQSKK---------------------T----------S-VENETG 28
PaWRI1          ----------MDTSSPLSNS---------------------ISSSSSSTTTTS-SSSSSS 28
AtWRI1          ----------MKK--RLTTS---------------------TCSSSPSSSVSS-STTTSS 26
                                                                  .

ZmWRI2          LSGELVPRRALALRKERVCTAKERISRMP-PCAAGKRSSIYRGVT--------------- 80
PaWRI2          -TDRLLFRGLKKARKERVCTAKERISKMP-PCAAGKRSSIYRGVT--------------- 90
AtWRI2          SDQMLLYRGFKKAKKERGCTAKERISKMP-PCTAGKRSSIYRGVTRFGCELGVVLSVLEY 90
ZmWRI1a         P---PGKRR-RAATAKAGAEPNKRIRKDPAAAAAGKRSSVYRGVT--------------- 69
ZmWRI3/4b       NAAVDLAAR-VKPKRTRKSVP---------RESPSQRSSVYRGVT--------------- 58
PaWRI3          D--NNSKST-TKVKRTRKSVP---------RESPTQRSSIYRGVT--------------- 45
AtWRI3          DDQSATSVV-LKAKRKRRSQP---------RDAPPQRSSVHRGVT--------------- 63
PaWRI1          PLSQ--PTK-TKIKRTRRN---LNPQKSQFGANPGRRSSVYRGVT--------------- 67
AtWRI1          PIQSEAPRP-KRAKRAKKSSPSGDKSHNPTSPASTRRSSIYRGVT--------------- 70
                                                    :***::****

ZmWRI2          --------------------RHRWTGRYEAHLWDKSTWNQNQN-KKGKQVYLGAYDDEEA 119
PaWRI2          --------------------RHRWTGRYEAHLWDKSTWNQNQN-KKGKQVYLGAYDDEEA 129
AtWRI2          GMFGLVDMHFVSRLKLEEMSRHRWTGRYEAHLWDKSTWNQNQN-KKGKQVYLGAYDDEEA 149
ZmWRI1a         --------------------RHRWTGRFEAHLWDKHCLAALHNKKKGRQVYLGAYDSEEA 109
ZmWRI3/4b       --------------------RHRWTGRFEAHLWDKNSWNESQN-KKGKQVYLGAYDDEDA 97
PaWRI3          --------------------RHRWTGRYEAHLWDKNCWNETQN-KKGRQ---GAYDDETA 81
AtWRI3          --------------------RHRWTGRYEAHLWDKNSWNETQT-KKGRQVYLGAYDEEDA 102
PaWRI1          --------------------RHRWTGRYEAHLWDKSSWNPVQN-KKGRQVYLGAYDDEEA 106
AtWRI1          --------------------RHRWTGRFEAHLWDKSSWNSIQN-KKGKQVYLGAYDSEEA 109
                                    *******:*******      :. ***:*   ****.* *

ZmWRI2          AARAYDLAALKYWGAGTQINFPVSDYARDLEEMQMISKEDYLVSLRRKSSAFYRGLPKYR 179
PaWRI2          AARAYDLAALKYWGPGTLINFPVSDYSRDLEEMQMVSREDYLASLRRKSSGFSRGFSKYR 189
AtWRI2          AARAYDLAALKYWGPGTLINFPVTDYTRDLEEMQNLSREEYLASLRRKSSGFSRGIAKYR 209
ZmWRI1a         AARAYDLAALKYWGPETLLNFPVEDYSSEMPEMEAVSREEYLASLRRRSSGFSRGVSKYR 169
ZmWRI3/4b       AARAYDLAALKYWGPDTILNFPASAYEAELKEMEGQSREEYIGSLRRKSSGFSRGVSKYR 157
PaWRI3          AAHAYDLAALKYWGQDTILNFPASTYEEELKEMEGQSKEEYIGSLRRKSSGFSRGVSKYR 141
AtWRI3          AARAYDLAALKYWGRDTILNFPLCNYEEDIKEMESQSKEEYIGSLRRKSSGFSRGVSKYR 162
PaWRI1          AAHTYDLAALKYWGPDTILNFPLNTYAKEFEEMQMVSKEEYLASLRRRSSGFSRGVSKYR 166
AtWRI1          AAHTYDLAALKYWGPDTILNFPAETYTKELEEMQRVTKEEYLASLRRQSSGFSRGVSKYR 169
                **::*********  * :***   *  :: **:  ::*:*: ****:**.* **. ***

ZmWRI2          GLLRQLHNSRWDTSLGL--GNDYM-S--LSCGKDIMLDGKFAGS----FGLERKIDLTNY 230
PaWRI2          GVSRP-QSSRWETSFGRILGNENFNSLNCSTSEDAATENEYTGT----FFLERKIDLTNY 244
AtWRI2          GLQ-----SRWDASASRMPGPEYFSNIHYGAGDDRGTEGDFLGS----FCLERKIDLTGY 260
ZmWRI1a         GVARHHHNGRWEARIGRVFGNKYLYLGTFDTQEEAAKAYDLAAIEYRGVNAVTNFDISCY 229
ZmWRI3/4b       GVARHHHNGRWEARIGRVFGNKYLYLGTYGTQEEAAMAYDMAAIEYRGLNAVTNFDLSRY 217
PaWRI3          GVARHHHNGRWEARIGRVFGNKYLYLGTYATQEEAATAYDMAAIEYRGLNAVTNFDLSRY 201
AtWRI3          GVAKHHHNGRWEARIGRVFGNKYLYLGTYATQEEAAIAYDIAAIEYRGLNAVTNFDISRY 222
PaWRI1          GVARHHHNGRWEARIGRVFGNKYLYLGTYSTQEEAAAAYDMAAIEYRGLNAVTNFDISHY 226
AtWRI1          GVARHHHNGRWEARIGRVFGNKYLYLGTYNTQEEAAAAYDMAAIEYRGANAVTNFDISNY 229
                *:       .**::  .    * . :         .:     .  .         ::*:: *

ZmWRI2          IRWWLPKKTRQSDTSK--------T-EEIAD-------------EIRAIES-SMQQTEPY 267
PaWRI2          IRWWGPKKIRRSDPITKSSDETHGI-SDAGS-------------ELKTFEW-PSQHTEPY 289
AtWRI2          IKWWGANKNRQPESSSKAS-EDANV-EDAGT-------------ELKTLEH-TSHATEPY 304
ZmWRI1a         LDHPLFLAQLQQEPQVVPALNQEPQPDQS----ETGTTEQEPESSEA---------KTP- 275
ZmWRI3/4b       IKWLRPGAGAA--------QNPHPMLDGLAQQLLL-----SPEGTIDGAAFHQQQHDHRQ 264
```

```
PaWRI3          IKWLRPGSQNPNSNT---DQNPNPSPSPS---TST-----SPNSDIGL----GFLHHHSS 246
AtWRI3          LKLPVPENPIDTANN--LLESPHSDLSPF-----I-----KPNHESDL----SQSQSSSE 266
PaWRI1          IRCQPPPPQPPQPSA--PFQ--------------------EP------------HHVAP- 251
AtWRI1          IDRLKKKGVFPFPVN--QANHQEGILVEAKQEVETREAKEEPREEVK-----QQYVEEP- 281
                :

ZmWRI2          KLPSLGFS----SPSKPSS------MGLSACSILSQSDAFKSFLEKSTKLSEECSLSKEI 317
PaWRI2          QLPSLGLS----CKGKPFA------SAMSACRILAQSAAFKKMQEKASEAQDGEHTNNI- 338
AtWRI2          KAPNLGVL----CGTQRKEKEISSPSSSSALSILSQSPAFKSLEEKVLKIQESCNNENDE 360
ZmWRI1a         --DGSAEPD---------E------------NAVPDDTAEPLTTVDDSIEEGLWS----- 307
ZmWRI3/4b       -QGAAELPL-------PPRASLGHTPTTSALGLLLQSSKFKEMIQRASAAESGTT----- 311
PaWRI3          -GGGDGVPL-------PRA-------GGGALNLLLQSTKFKEMLERTSAVDS-------- 283
AtWRI3          -DN------------------------DDRKTKLLKSSPL-----VAEEVIG-------- 288
PaWRI1          --PQIQIP-----------ENSG----------PIDQTAI-----MDPLTDQPWN----- 278
AtWRI1          --PQEEEEKEEEKAEQQEAEIVGYSEEAAVVNCCIDSSTIMEMDRCGDNNELAWN----- 334
                                                   ..

ZmWRI2          VEGKTVASVPATG--------YDTGAININMNELLVQRSTYSMAPVMPTPMKSTWSPADP 369
PaWRI2          GHEKTVLKLSSVGGSE------SSGVGL-SLGEMPLQKTSYPLGPFLSAPLLTNCSNIDP 391
AtWRI2          NANRNIINMEKNNGKAIEKPVVSHGVALGGAAALSLQKSMYPLTSLLTAPLLTNYNTLDP 420
ZmWRI1a         ---------------------------------PCMDYELDTMSR----PN-------FG 323
ZmWRI3/4b       ---------------------------------TVTTTSSSSSQPPTPTPTPSPSPPPTP 338
PaWRI3          -----------------------------------------------------------P 284
AtWRI3          -----------------------------------------------------------P 289
PaWRI1          ---------------------------------LCMDP--TFNS--IPVPNIA----LDK 297
AtWRI1          ---------------------------------FCMMD--TGFSPFLTDQNLA----NEN 355

ZmWRI2          SVDP----------------------------------LFWSNFVLPSSQPVTMATITTT 395
PaWRI2          SPDS----------------------------------AFWTNLIQPTGLSLSTT----- 412
AtWRI2          LADP----------------------------------ILWTPFLPSGSSLTSEV----- 441
ZmWRI1a         SSINLSEWFADADFDCNIGCLFDGCSAADEGS---KDGVGLADFSLFEAGDVQ--LKD-- 376
ZmWRI3/4b       PVQPARDASPQCSFPEDIQTFFGCEDVAGVGAGVDVDALFFGDLAARMELSRHLRTKKMV 398
PaWRI3          STTPEADNPPRCSFPDDIQTYFECEDPGV------GDDVIFGDLNSFAAPLIEGEWD--- 335
AtWRI3          S-TPPEIAPPRRSFPEDIQTYFGCQNSGKLTA--EEDDVIFGDLDSFLTPDFYSELNDC- 345
PaWRI1          SI-ELHDLFNGTGFEDNIEFLFDGAEGG------------------FPTGPTTTGIHECE 338
AtWRI1          PI-EYPELFNELAFEDNIDFMFDDGKHE------------------CLNL---ENLDCCV 393

ZmWRI2          TFA-------------------------KNEVSSSDPFQSQE------------------ 412
PaWRI2          -HR-------------------------KNEISSTYTFFRQE------------------ 428
AtWRI2          -TK-------------------------TETSCSTYSYLPQEK----------------- 458
ZmWRI1a         --------------------------VLSDME-----------------EGIQPPAMISV 393
ZmWRI3/4b       RHTKQERQQNRQTAPSLF------------------------------------------ 416
PaWRI3          ------------------------------------------------------------ 335
AtWRI3          ------------------------------------------------------------ 345
PaWRI1          GNE-MK-----LDD-GIFENSGGFEGDFLNDIEEGGECGLIVENMNASVDSISYPLPISI 391
AtWRI1          VGRESP-----PSSSSPLSCLSTDSASSTTTTTTSVSCNYLV------------------ 430

ZmWRI2          --        412
PaWRI2          --        428
AtWRI2          --        458
ZmWRI1a         CN        395
ZmWRI3/4b       --        416
PaWRI3          --        335
AtWRI3          --        345
PaWRI1          CS        393
AtWRI1          --        430
```

Supplemental Figure 3. Multiple sequence alignment of WRI1 homologs from the select three species.

The alignment shows KIN10 target sites (red box) in the WRI1 homologs with reference to T70 and S166 identified in *At*WRI1. The S166 residue is mutated in *At*WRI2 and *Zm*WRI2. The highlighted amino acid residues correspond to the PEST motifs.

Supplementary Figure 4.

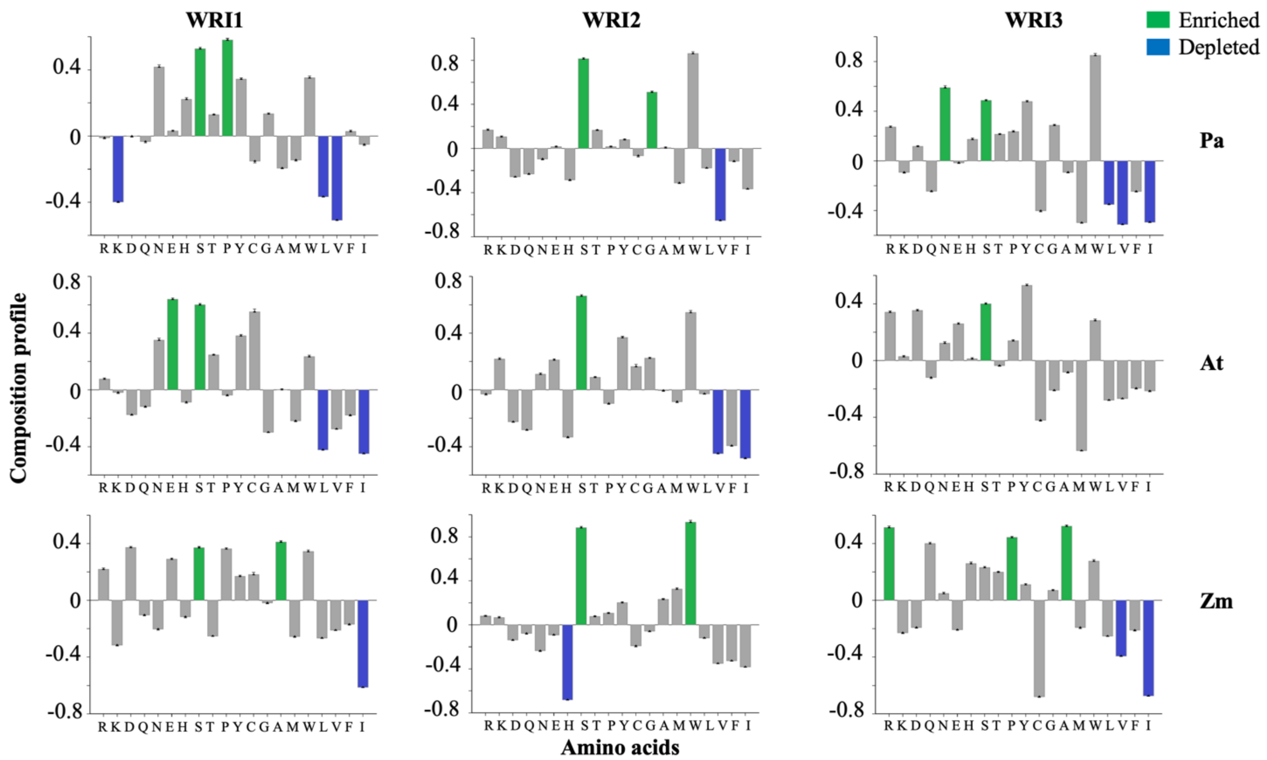

Supplementary Figure 4: Amino acid composition profile of WRI1 homologs.

Amino acid composition profile of WRI1 homologs showing the enriched and depleted amino acids as compared to SwissProt51 database. The amino acids are arranged in the increasing order of hydrophobic character on the x-axis and the y-axis represents the composition profile. The values >0 represents an enrichment and <0 represents a depletion of corresponding amino acids in the protein sequence. The significantly ($p<0.05$) enriched (green) and depleted (blue) residues are denoted in color.

Supplementary Figure 5.

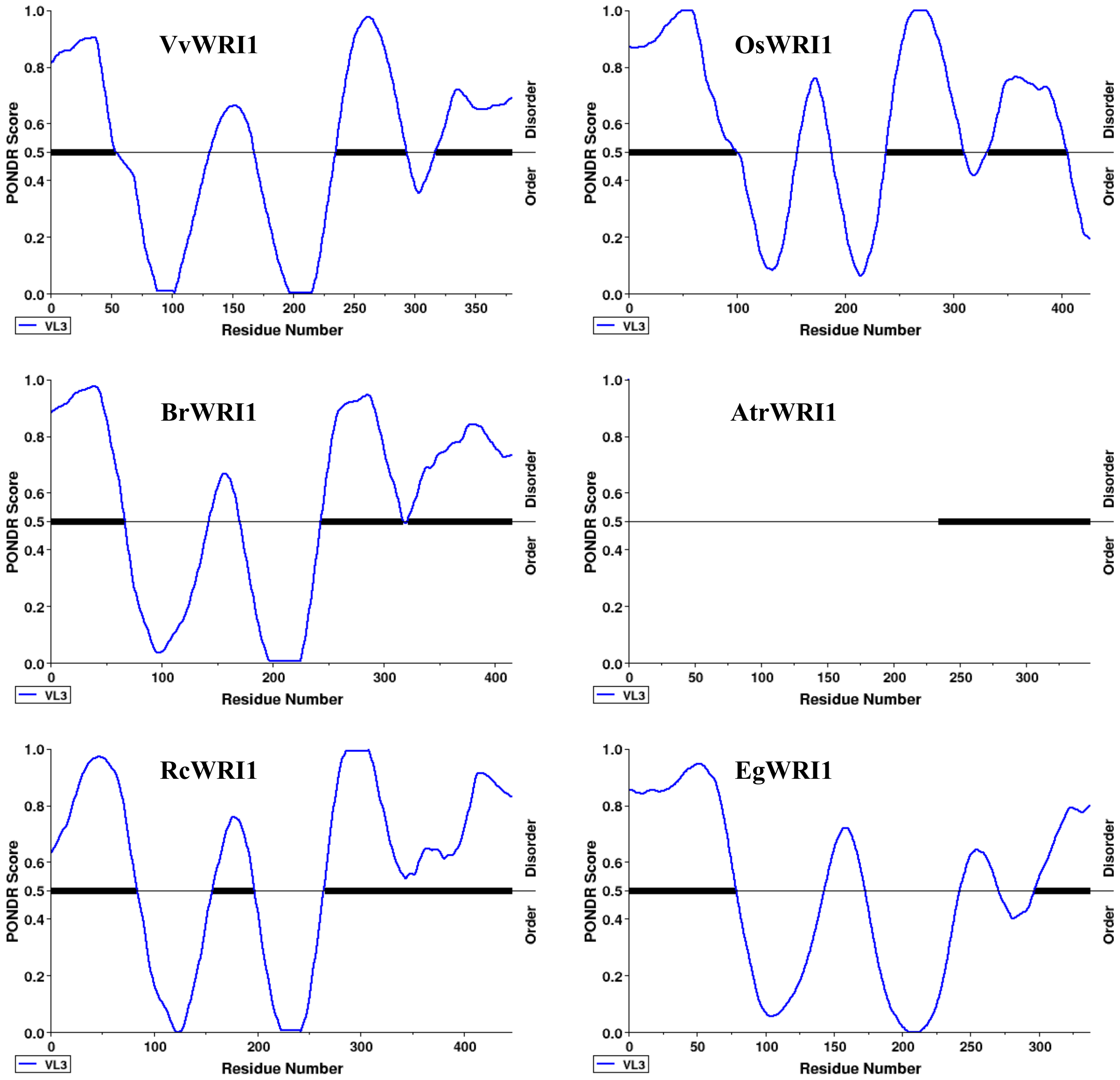

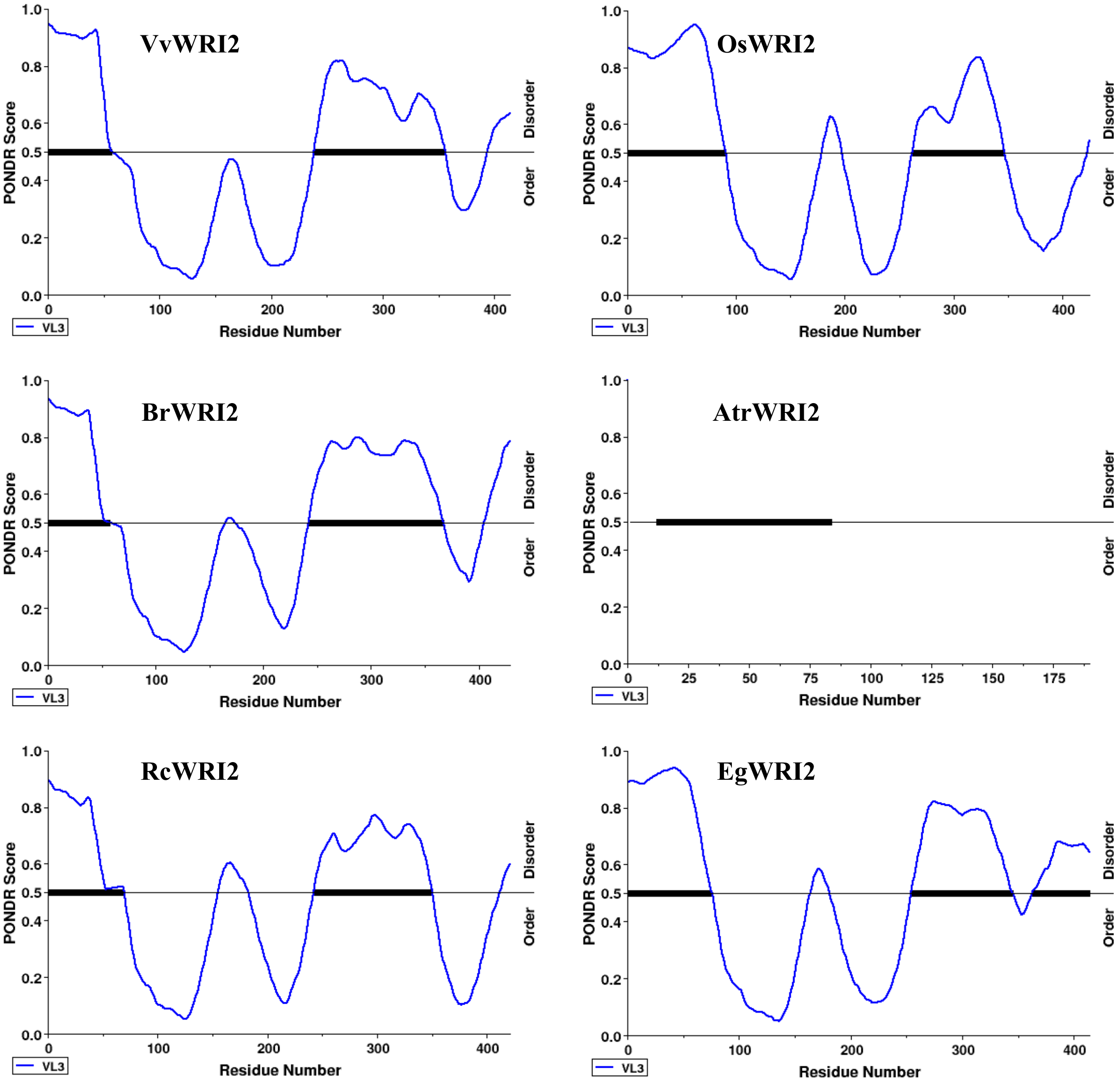
VvWRI2
OsWRI2
BrWRI2
AtrWRI2
RcWRI2
EgWRI2
PONDR Score
Residue Number
Disorder
Order
VL3
1.0
0.8
0.6
0.5
0.4
0.2
0.0
0
100
200
300
400
25
50
75
125
150
175

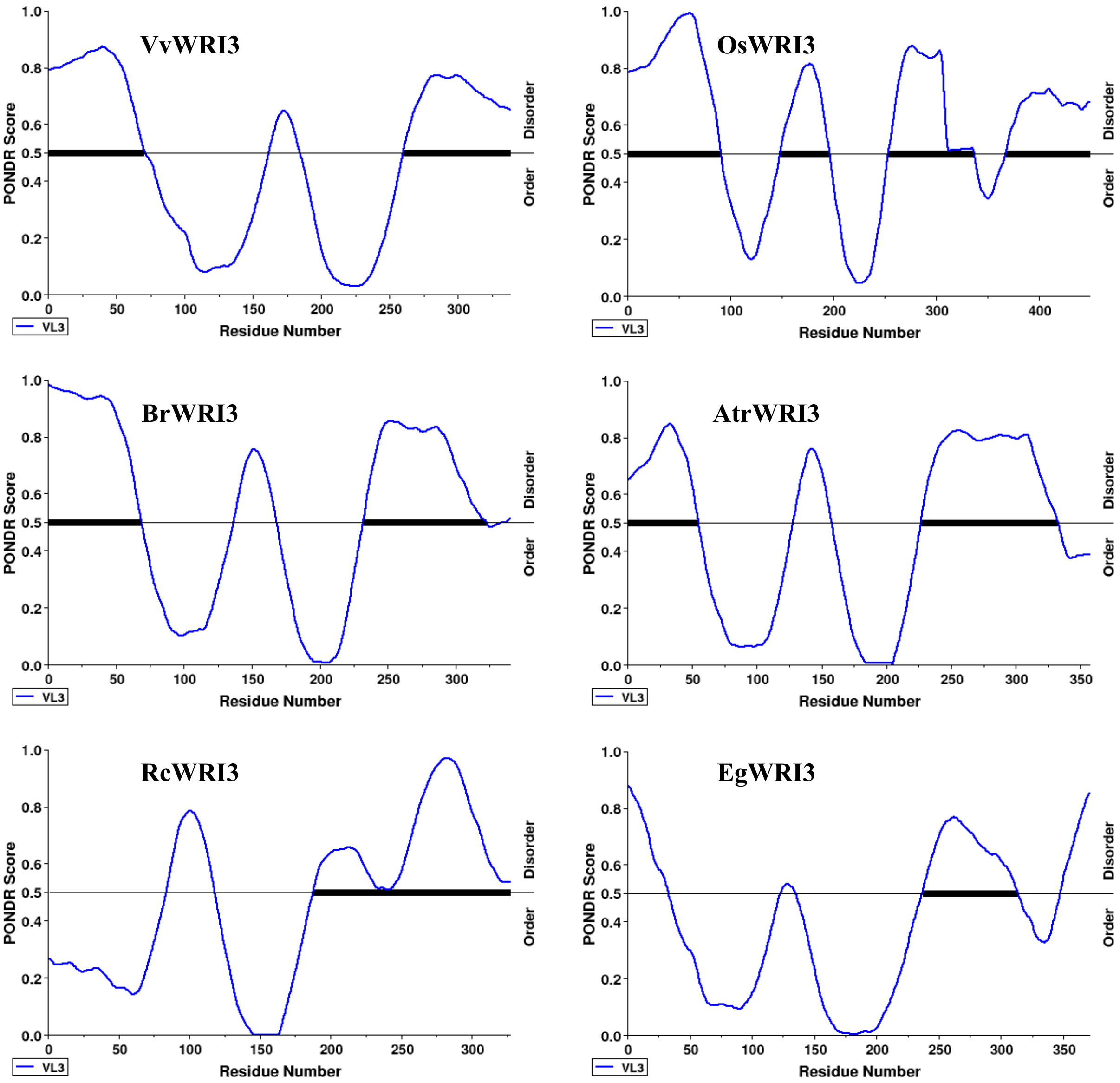

Supplementary Figure 5: **Graphical representation of the predicted IDRs**

The predicted IDRs in all the WRI homologs are graphically plotted with the amino acid position on the x-axis and the PONDR score on the y-axis. Regions showing PONDR score >0.5 are considered as IDRs. For *Atr*WRI1 and *Atr*WRI2, although the figures are not showing the PONDR score, the predicted IDRs by the same software are as follows: 30-53, 137-160, 234-348 for WRI1 and 12-84, 168-190 for WRI2. Both of them have the *C*-terminal IDR.
